# RNase L activation in the cytoplasm induces aberrant processing of mRNAs in the nucleus

**DOI:** 10.1371/journal.ppat.1010930

**Published:** 2022-11-01

**Authors:** James M. Burke, Nina Ripin, Max B. Ferretti, Laura A. St Clair, Emma R. Worden-Sapper, Fernando Salgado, Sara L. Sawyer, Rushika Perera, Kristen W. Lynch, Roy Parker

**Affiliations:** 1 Department of Biochemistry, University of Colorado Boulder, Boulder, Colorado, United States of America; 2 Department of Molecular Medicine, University of Florida Scripps Biomedical Research, Jupiter, Florida, United States of America; 3 Howard Hughes Medical Institute, University of Colorado Boulder, Boulder, Colorado, United States of America; 4 Department of Biochemistry and Biophysics, Perelman School of Medicine, University of Pennsylvania, Philadelphia, Pennsylvania, United States of America; 5 Center for Vector-Borne Infectious Diseases, Department of Microbiology, Immunology and Pathology, Colorado State University, Fort Collins, Colorado, United States of America; 6 Center for Metabolism of Infectious Diseases, Colorado State University, Fort Collins, Colorado, United States of America; 7 Department of Molecular, Cellular and Developmental Biology, University of Colorado Boulder, Boulder, Colorado, United States of America; 8 BioFrontiers Institute, University of Colorado Boulder, Boulder, Colorado, United States of America; Fundación Instituto Leloir-CONICET, ARGENTINA

## Abstract

The antiviral endoribonuclease, RNase L, is activated by the mammalian innate immune response to destroy host and viral RNA to ultimately reduce viral gene expression. Herein, we show that RNase L and RNase L-mediated mRNA decay are primarily localized to the cytoplasm. Consequently, RNA-binding proteins (RBPs) translocate from the cytoplasm to the nucleus upon RNase L activation due to the presence of intact nuclear RNA. The re-localization of RBPs to the nucleus coincides with global alterations to RNA processing in the nucleus. While affecting many host mRNAs, these alterations are pronounced in mRNAs encoding type I and type III interferons and correlate with their retention in the nucleus and reduction in interferon protein production. Similar RNA processing defects also occur during infection with either dengue virus or SARS-CoV-2 when RNase L is activated. These findings reveal that the distribution of RBPs between the nucleus and cytosol is dictated by the availability of RNA in each compartment. Thus, viral infections that trigger RNase L-mediated cytoplasmic RNA in the cytoplasm also alter RNA processing in the nucleus, resulting in an ingenious multi-step immune block to protein biogenesis.

## Author summary

Ribonuclease L (RNase L) initiates rapid and widespread degradation of cellular mRNAs in response to double-stranded RNA or viral infection. How the mass degradation of mRNAs affects cellular functions is unknown. Herein, we show that RNase L-mediated mRNA decay is predominately localized to the cytoplasm. Consequently, RNA-binding proteins re-localize from the cytoplasm to the nucleus upon RNase L activation. Coinciding with the influx of RNA-binding proteins into the nucleus, we also observe perturbation of nuclear processing of mRNAs, and these processing defects down-regulate key antiviral cytokines, such as type I interferons. Importantly, we observe these processes occurring during dengue virus and SARS-CoV-2 infection, revealing previously unknown aspects of antiviral gene regulation during pathogenic viral infections. Combined, these findings uncover novel mechanisms by which RNase L regulates host gene expression and expand the potential means by which RNase L blocks protein synthesis.

## Introduction

Ribonuclease L (RNase L) is an endoribonuclease that limits the replication of diverse viruses, including influenza virus, chikungunya virus, SARS-CoV-2, and vaccinia virus in mammals [1–4]. RNase L is activated by 2’-5’-oligo(A), which is produced by 2’-5’-oligoadenylate synthetases (OASs) upon binding to viral or endogenous dsRNA reviewed in [5,6]. RNase L cleaves at UN^N motifs in single-stranded RNA (ssRNA) regions [7,8], and destruction of viral RNAs by RNase L reduces viral replication [2,9,10]. In addition, RNase L also cleaves several types of cellular RNAs, including rRNAs, tRNAs, and mRNAs [8,11–14].

We, and others, recently demonstrated that RNase L activation results in widespread degradation of host mRNAs [13,14]. This has multiple impacts to cellular RNA biology. First, it allows for antiviral programming of translation since several immediate early antiviral mRNAs (i.e., *interferon* mRNAs) escape RNase L-mediated mRNA decay. Second, it inhibits the assembly of stress granules [15], cytoplasmic ribonucleoprotein (RNP) complexes proposed to modulate aspects of the antiviral response [16–18]. Third, it promotes the formation of an alternative cytoplasmic RNP granule of unknown function termed RNase L-dependent bodies (RLBs) [15]. Fourth, it inhibits nuclear mRNA export, which substantially reduces protein production of influenza virus as well as host cytokines induced by dsRNA, dengue virus infection, or SARS-CoV-2 infection [19,20]. Lastly, it results in the translocation of poly(A)-binding protein (PABP) from the cytoplasm to the nucleus [13].

Similar to RNase L-mediated mRNA decay, cellular mRNAs are reduced by Kaposi’s sarcoma-associated herpesvirus (KSHV) and murine gammaherpesvirus 68 (MHV68) via the SOX and muSOX proteins, respectively, which results in the translocation of host RNA-binding proteins, including PABP, to the nucleus [21–25]. Moreover, SOX/muSOX-mediated translocation of PABP to the nucleus results in hyperadenylation of host mRNAs, inhibition of host mRNA export, and inhibition of transcription of host genes [22–26]. Therefore, we asked whether RNase L-mediated RNA decay resulted in RNA-binding protein influx into the nucleus and how that affects antiviral gene induction in response to dsRNA or infection with either dengue virus or severe acute respiratory syndrome coronavirus 2 (SARS-CoV-2).

Herein, we demonstrate that RNase L-mediated degradation of host mRNAs primarily occurs in the cytoplasm, which leads to the re-localization of many RBPs to the nucleus in a manner dependent on intact nuclear RNA. This demonstrates the principle that the distribution of RBPs between subcellular compartments is dependent on the availability of their binding sites. The influx of RBPs into the nucleus is concurrent with global RNase L-dependent RNA processing alterations including alternative splicing, intron retention and transcription termination defect leading to downstream of gene (DoG) transcript production, which correlate with inhibition of nuclear export of *IFN* mRNAs. These are general responses since we observe these alterations in RNA processing occur during exposure to exogenous dsRNA, dengue virus infection, or SARS-CoV-2 infection. These findings show that RNase L-mediated mRNA decay alters the balance of RNA-binding protein subcellular localization, host RNA processing events, and antiviral gene expression.

## Results

### RNase L activation triggers re-localization of multiple RNA-binding proteins to the nucleus

Many RNA-binding proteins (RBPs) shuttle between the nucleus and the cytoplasm, and cellular stresses are known to modulate the location of several RBPs [27,28]. For example, many cytoplasmic RBPs (i.e., G3BP1 and PABPC1) and nuclear RBPs (i.e., HuR and TIA1) accumulate in cytoplasmic ribonucleoprotein complexes termed stress granules upon oxidative, osmotic, or endoplasmic reticulum stresses [29,30]. This re-localization of RBPs is thought to be the result of increased availability of RNA-binding sites and/or changes in RNA abundance [31].

RNase L degrades RNA and inhibits stress granules [13]. Concurrently, RNase L promotes the formation of stress granule-like ribonucleoprotein complexes termed RNase L-dependent bodies (RLBs). RNase L-dependent bodies can be distinguished from stress granules by being smaller, more spherical, and having a different protein composition [15]. Thus, we asked whether RNase L activation alters the localization of RBPs with respect to the cytoplasm and nucleus, similar to what has been seen when mRNAs are degraded during herpesvirus infection [21–25].

**Fig 1:**
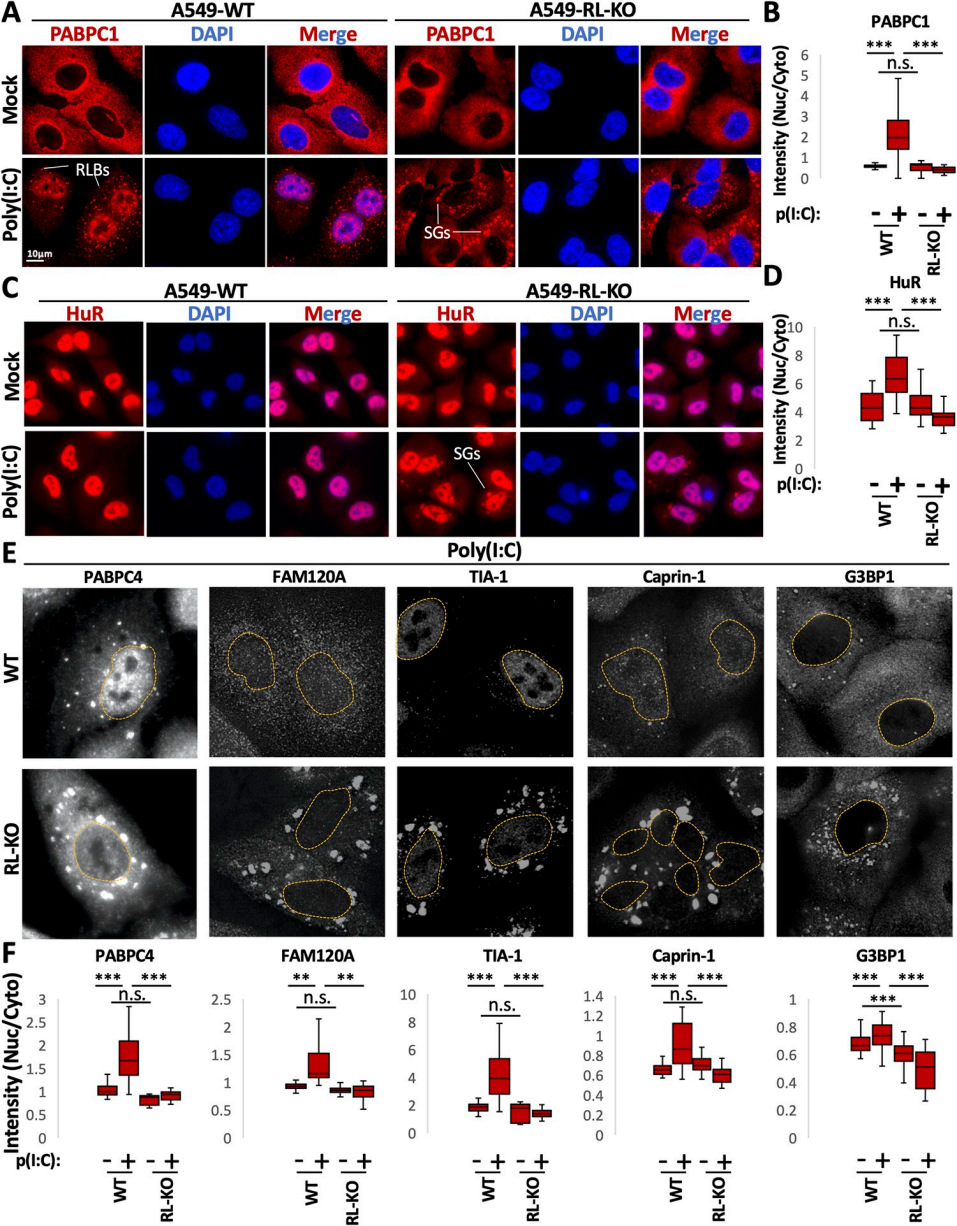
RNase L activation increases the concentration of RBPs in the nucleus. (A) Immunofluorescence assay for PABPC1 in wild-type (WT) or RNase L knockout (RL-KO) A549 cells following four hours of lipofection with or without poly(I:C). (B) Quantification of the nuclear to cytoplasmic ratio of PABPC1 intensity as represented in (A). Between 56–118 cells were analyzed from three independent replicates. (B and C) Similar to (A and B) but for HuR. Between 15–22 cells were analyzed (E) Panels show indicated RBP immunofluorescence, with the nucleus outlined, in WT and RL-KO cells following transfection with poly(I:C). (F) Quantification of the nuclear to cytoplasmic ratio of immunofluorescence intensity of indicated RNA-binding proteins in WT and RL-KO cells transfected with or without poly(I:C), as represented in (E). Between 30–127 cells were counted from three independent replicates for G3BP1 an TIA-1, two replicates for FAM120A and Caprin-1, and one replicate for PABPC4. Note, only WT cells with RLBs or RL-KO cells with stress granules were analyzed in the poly(I:C)-treated cells since not all cells responds to poly(I:C). Images of PABPC4 and TIA during mock conditions can be observed in Fig 3. Images of FAM120A, Caprin-1, and G3BP1 are shown in S1A–S1C Fig. Statistical significance was determined using a one-way ANOVA with Tukey’s HSD. * is p < 0.05, ** is p < 0.01, *** is p < 0.001. Statistical analyses between the most relevant comparisons are shown the graphs. Comparisons between all groups are included in S1 Data.

We transfected parental (WT) or RNase L-KO (RL-KO) A549 cells with or without poly(I:C), a double-stranded viral RNA mimic that initiates the OAS/RNase L, RLR-MAVS, and PKR innate immune response pathways. We first performed immunofluorescence assays for PABPC1, which we previously determined translocates to the nucleus in an RNase L-dependent manner [13]. Consistent with our previous observations, PABPC1 primarily localized to the cytoplasm during mock conditions in both WT and RL-KO cells (Fig 1A and 1B). Upon lipofection of poly(I:C) in RL-KO cells, PABP accumulated in cytoplasmic stress granules (Fig 1A and 1B). However, poly(I:C) transfection in WT cells resulted in the translocation of PABPC1 to the nucleus, as well as the localization of PABPC1 to RNase L-dependent bodies (Fig 1A).

We next analyzed HuR, which primarily localizes to the nucleus in unstressed cells (Fig 1C and 1D). In WT cells lipofected with poly(I:C), we observed a significant increase in the nuclear to cytoplasmic ratio of HuR (Fig 1C and 1D). However, RL-KO cells transfected with poly(I:C) displayed a reduction in the nuclear to cytoplasmic ratio of HuR, consistent with its accumulation in SGs (Fig 1C and 1D). Thus, regardless of whether an RBP is localized to the cytoplasm (PABPC1) or nucleus (HuR) under non-stress conditions, RNase L activation during dsRNA stress promotes the accumulation of RBPs in the nucleus.

One possibility is that the inhibition of SGs results in RBP re-localization to the nucleus during stress. To examine this, we performed IF on additional RBPs that typically localize to SGs in WT and RL-KO cells transfected with or without poly(I:C). A striking result was that most RBPs we examined translocated to the nucleus in a RNase L-dependent manner in response to poly(I:C), including PABPC4, FAM120A, TIA-1, Caprin-1, and G3BP1 (Figs 1E,1F and S1). Moreover, we observed an increase in the nuclear to cytoplasmic ratio of CUGBP-1, Pumilio, FMRP, and Ataxin-2 in WT cells in comparison to RL-KO cells during poly(I:C) stress (S1 Fig). These data indicate that RNase L activation results in the accumulation of RBPs in the nucleus during dsRNA stress. This is similar to earlier results when cytosolic mRNAs are degraded during herpesvirus infection [22,23,25,26]. Thus, our data support that general widespread degradation of cytosolic RNA leads to increased accumulation of specific RBPs in the nucleus.

### RNase L and RNase L-mediated RNA decay are primarily localized to the cytoplasm

**Fig 2:**
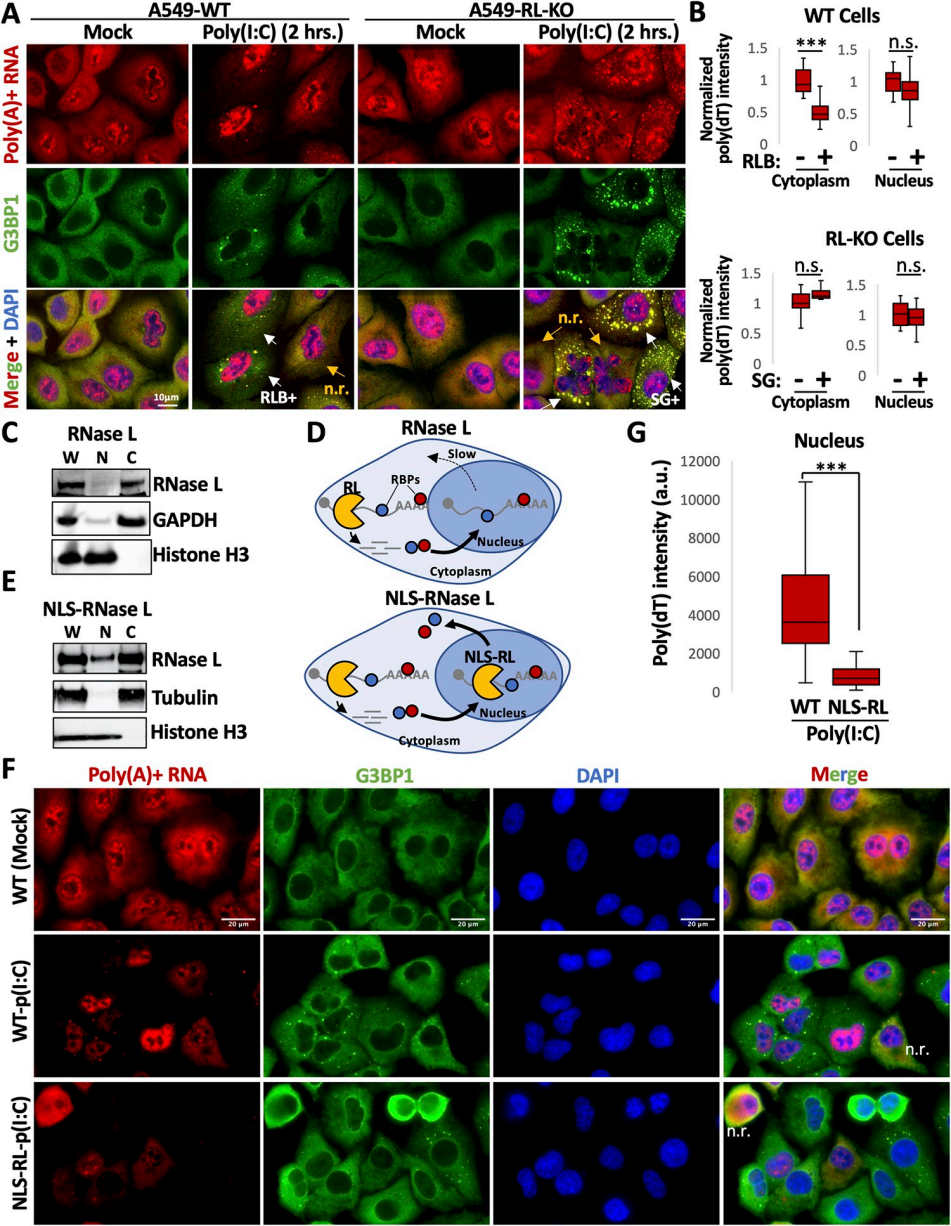
RNase L-mediated decay of RNAs specifically occurs in the cytoplasm. (A) FISH for poly(A)+ RNA and immunofluorescence assay for G3BP1 in WT and R-KO A549 cells. WT cells with RNase L-dependent bodies (RLBs) or RL-KO cells containing stress granules (SGs) are indicated by white arrows. Cells without RLBs or SGs are non-responsive (n.r.) and are indicated by yellow arrows. (B) Quantification of poly(A)+ RNA signal in the nucleus (nuc.) or cytoplasm (cyto.) in WT and RL-KO cells with or without RLBs or SGs, respectively, as represented in (A). Between 18–35 cells from at least three fields of view were analyzed. (C) Immunoblot analysis of endogenous RNase L in whole cell lysate (w), nuclear fraction (n), or cytoplasmic fraction (c) from A549-WT cells. (D) Schematic showing RBP re-localization following either cytoplasmic RNase L activation (left) or activation of nuclear-localized RNase L (RL-NLS). (E) Immunoblot for RNase L in whole cell (w), nuclear (n), and cytoplasmic (c) crude fractions showing nuclear localization of the RNase L-NLS. (F) Immunofluorescence assay for G3BP1 and FISH for poly(A)+ RNA in parental A549 cells or A549 that express NLS-RNase L construct following mock or poly(I:C) lipofection. N.r. indicates cells not responding to poly(I:C) based on the lack of RLBs marked by G3BP1 and poly(A)+ RNA, and the absence of reduced cytoplasmic poly(A)+ RNA staining. (G) Quantification of mean poly(A)+ RNA signal intensity (arbitrary units, a.u.) in WT or WT-RL-NLS cells treated with poly(I:C) as represented in (F). Between 92–110 cells from at least ten fields of view were analyzed for each group. Statistical significance was determined using a one-way ANOVA with Tukey’s HSD. * is p < 0.05, ** is p < 0.01, *** is p < 0.001. Statistical analyses between the most relevant comparisons are shown the graphs. Comparison between all groups are included in S1 Data.

One mechanism by which RNase L could alter the localization of RBPs between the nucleus and cytoplasm is differential RNA degradation between the cytoplasm and nucleus, whereby higher RNA decay in the cytoplasm relative to the nucleus would lead to disassociation of RBPs from cytoplasmic RNA more rapidly than from nuclear RNA. To assess this, we quantified poly(A)+ RNA in the cytoplasm and nucleus of WT and RL-KO cells lipofected with or without post-poly(I:C) (Fig 2A). We also stained cells for G3BP1 to identify cells undergoing a dsRNA response, whereby WT cells with activated RNase L contain RNase L-dependent bodies (RLBs) or RL-KO cells with activated PKR contain stress granules (SGs) [15].

Importantly, we did not observe a significant reduction of poly(A)+ RNA staining in the nucleus of WT cells that activated RNase L (RLB-positive) (Fig 2A and 2B), whereas poly(A)+ RNA was significantly reduced in the cytoplasm (Fig 2A and 2B). As expected, we did not observe reduced cytoplasmic poly(A)+ RNA staining in SG-positive RL-KO cells. Consistent with these observations, the *GAPDH* mRNA accumulates in the nucleus while being degraded in the cytoplasm when RNase L is activated [20]. These data indicate that RNase L-mediated RNA decay primarily occurs in the cytoplasm in A549 cells.

While previous studies have shown that RNase L can localize to both the cytoplasm and nucleus [32,33], we observed that endogenous RNase L is almost exclusively localized to the cytoplasm in A549 cells via cellular fractionation followed by immunoblot analysis (Fig 2C). Combined, these data indicate that RNase L-mediated RNA decay primarily occurs in the cytoplasm.

### Nuclear influx of RBPs is dependent on nuclear RNA

In principle, RNase L activation could alter RBP localization in two manners. First, RBPs may be subject to post-translational modifications, indirectly promoted by RNase L activation, that increase their nuclear accumulation. Alternatively, free RBPs may shuttle between the nucleus and cytosol faster than RBPs bound to RNA, and therefore the degradation of bulk cytoplasmic RNA would allow RBPs to shuttle to the nucleus, where binding to nuclear RNA would retain the RBPs in the nucleus. A prediction of this latter model is that the accumulation of nuclear RBPs will be dependent on the presence of a pool of nuclear RNA to bind the RBPs and increase their dwell time in the nucleus (Fig 2D).

To test whether nuclear RNA is required for RBP accumulation in the nucleus, we assayed RBP localization when nuclear RNAs are degraded concurrently with cytoplasmic RNAs in response to RNase L activation (Fig 2D). To do this, we used A549 cells that constitutively express RNase L tagged with a nuclear localization signal (NLS) that we previously generated and termed NLS-RNase L [34]. Unlike RNase L, which strictly localizes to the cytoplasm (Fig 2C), the NLS-RNase L localizes to both the cytosol and nucleus (Fig 2E). Measurement of poly(A)+ RNA via FISH confirmed that NLS-RNase L degrades nuclear RNAs in response to poly(I:C) lipofection (Fig 2F and 2G), which was not observed in untransfected cells (Fig 2B), consistent with our previous findings [34].

**Fig 3:**
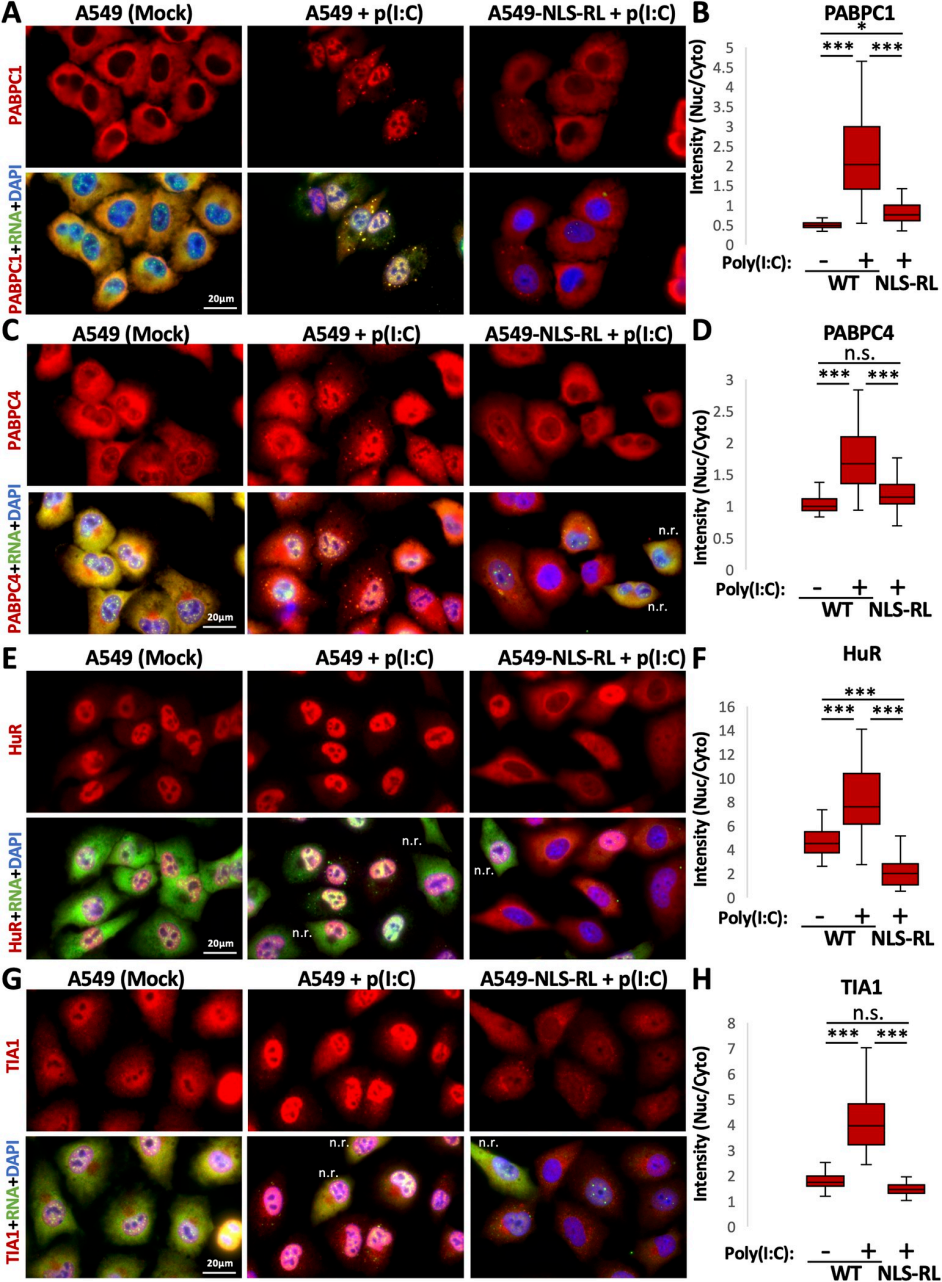
RNase L-mediated re-localization of RBPs to the nucleus is dependent on intact nuclear RNA. (A) Immunofluorescence for PABPC1 and FISH for poly(A)+ RNA in WT or WT cells expressing NLS-RNase L (NLS-RL) eight hours post-poly(I:C) transfection. (B) Quantification of the nuclear:cytoplasmic ratio of PABPC1 in mock-treated WT cells or WT or NLS-RL cells transfected with poly(I:C) as represented in (A). Between 48–70 cells from greater than six fields of view and two independent replicates were analyzed. (C-H) Similar analysis as (A) and (B) but for PABPC4 (C and D), HuR (E and F), or TIA1 (G and H). N.r. indicates cells that lack RLBs (G3BP1 foci) and did not reduce cytoplasmic poly(A)+ RNA levels, indicative of these cells not activating RNase L in response to poly(I:C), thus serving as internal controls. Statistical significance was determined using a one-way ANOVA with Tukey’s HSD. * is p < 0.05, ** is p < 0.01, *** is p < 0.001.

Strikingly, RNase L-mediated degradation of both nuclear and cytoplasmic RNA reduced the levels of PABPC1, PABPC4, HuR, and TIA1 in the nucleus in response to poly(I:C) (Fig 3A–3H). Specifically, in NLS-RNase L cells that displayed a reduction of poly(A)+ RNA staining in both the nucleus and cytoplasm following poly(I:C) lipofection, PABPC1, PABPC4, HuR, and TIA1 displayed reduced localization to the nucleus in comparison to WT cells that activated RNase L-mediated decay only in the cytoplasm following poly(I:C) lipofection (Fig 3A–3H). These data indicate that RNase L activation can initiate decay of nuclear RNAs when it is localized to the nucleus, and this in turn reduces the ability of RBPs to localize to the nucleus.

Thus, RNase L activation increases the nuclear localization of several RBPs by increasing the relative number of RBP-binding sites in the nucleus relative to the cytoplasm because of degradation of cytoplasmic but not nuclear mRNAs.

### RNase L activation alters host mRNA processing during the dsRNA response

The above data imply that RBPs translocated to the nucleus upon RNase L activation associate with RNA. This influx of RBPs would be predicted to compete for RNA-binding with RBPs involved in mRNA processing and thereby alter nuclear RNA processing. To examine if RNase L activation alters RNA processing, we analyzed high-throughput RNA sequencing (RNA-seq) of WT and RL-KO cells following six hours of either mock treatment or poly(I:C) lipofection [13].

To examine if there were changes to alternative splicing, we utilized MAJIQ (see methods) which identifies changes in alternative splicing patterns. Splicing changes are expressed for a given splicing event as the Percentage Spliced In (PSI). Thus, we compared the PSI of splicing events between WT or RL KO cells treated with or without poly(I:C) to identify changes in RNA splicing that occur in response to dsRNA and that are dependent on RNase L.

**Fig 4:**
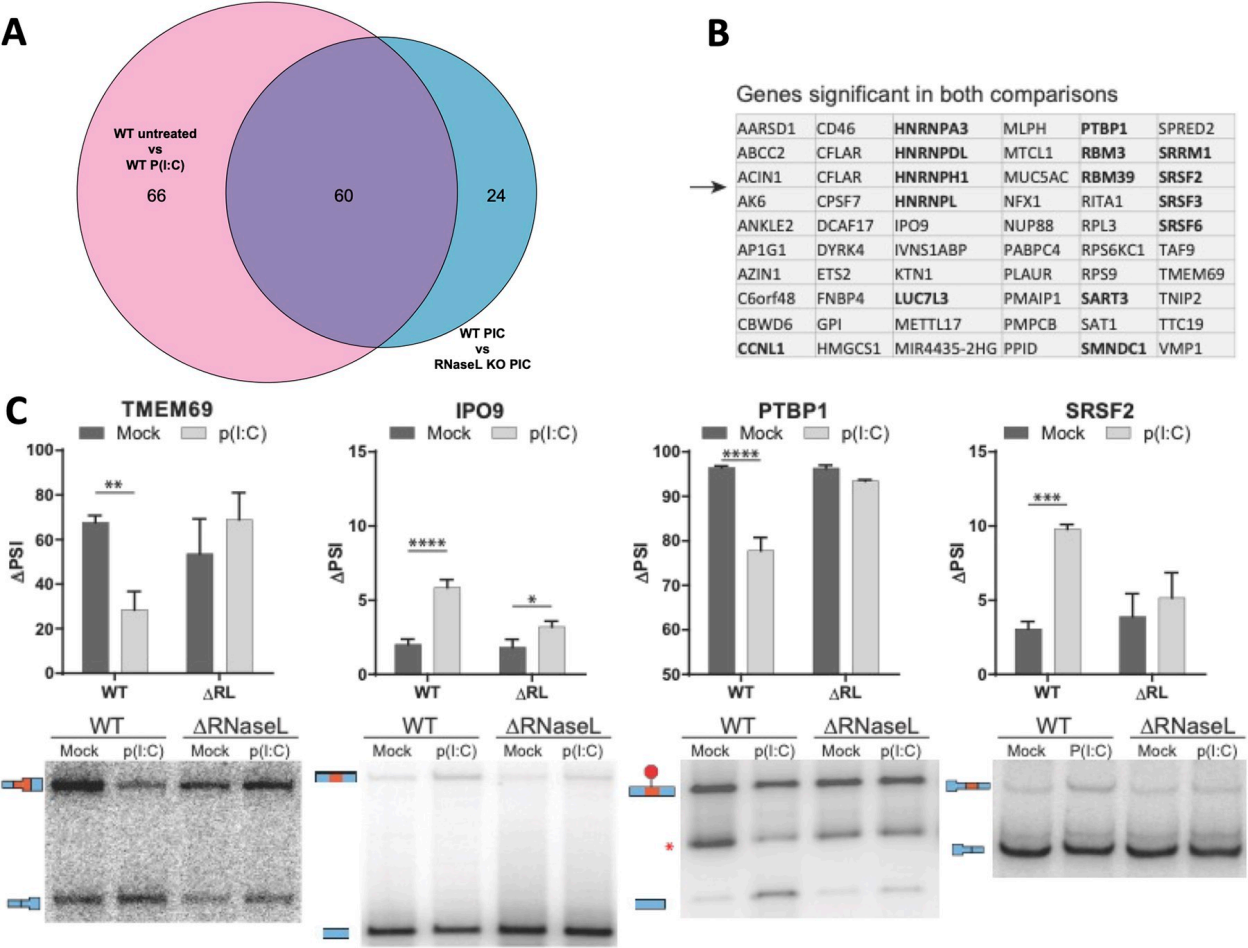
RNase L activation results in alterations to alternative splicing. (A) Venn diagram comparing significant ΔPSI values in RL KO vs WT cells treated with poly(I:C) and WT control vs WT poly(I:C) treated cells. (B) List of genes significant in both comparisons from panel A. RBPs known to regulate splicing are shown in bold. (C) Validation of splicing events by low cycle radiolabeled RT-PCR. The red asterisk in the PTBP1 gel is a nonspecific product. Bar graphs show quantification of four biological replicates. * is p < 0.05, ** is p < 0.01, *** is p < 0.001, *** is p < 0.0001. Holm-Sidak was used to correct for multiple t-tests using an alpha of 0.05.

This analysis identified 140 splicing events, across 136 genes, that showed differential splicing either due to poly(I:C) treatment in the WT cells, or were different between the WT and RL-KO cell lines post-poly(I:C). These changes are calculated as the difference in PSI between two conditions, the ΔPSI. The changes in splicing in both cases were generally correlated, which indicates that the majority of changes in alternative splicing observed are due to activation of RNase L (Fig 4A). Moreover, poly(I:C) lipofection in RL-KO cells resulted in only a small number of splicing alterations in comparison to mock treatment (S2 Fig). Splicing alteration in 60 genes were statistically significant under both comparisons (Fig 4B). Strikingly, 14 of these 60 genes encode RBPs involved in pre-mRNA splicing. This is notable since many RBPs autoregulate their own splicing [35], and this would be consistent with increased nuclear occupancy of RBPs following activation of RNase L.

To validate the analysis of RNA-Seq data, we prepared RNA from mock and poly(I:C) treated WT and RL-KO cells and examined a subset of splicing events by low cycle radiolabeled RT-PCR. In all four cases examined (TMEM69, IPO9, PTBP1, and SRSF2), we observed a change in exon inclusion with poly(I:C) treatment that was dependent on RNase L (Fig 4C). This demonstrates that RNase L activation can lead to changes in alternative splicing.

Another alteration of splicing can be overall decreased splicing rates and the increased retention of introns (intron retention (IR)). To determine if intron retention is affected by RNase L activation, we calculated an intron/exon ratio (TPM normalized intron over TPM normalized exon counts) per RNA for WT and RL-KO cells with or without poly(I:C) transfection. An increase in intron retention correlates with an increase in the intron/exon ratio.

**Fig 5:**
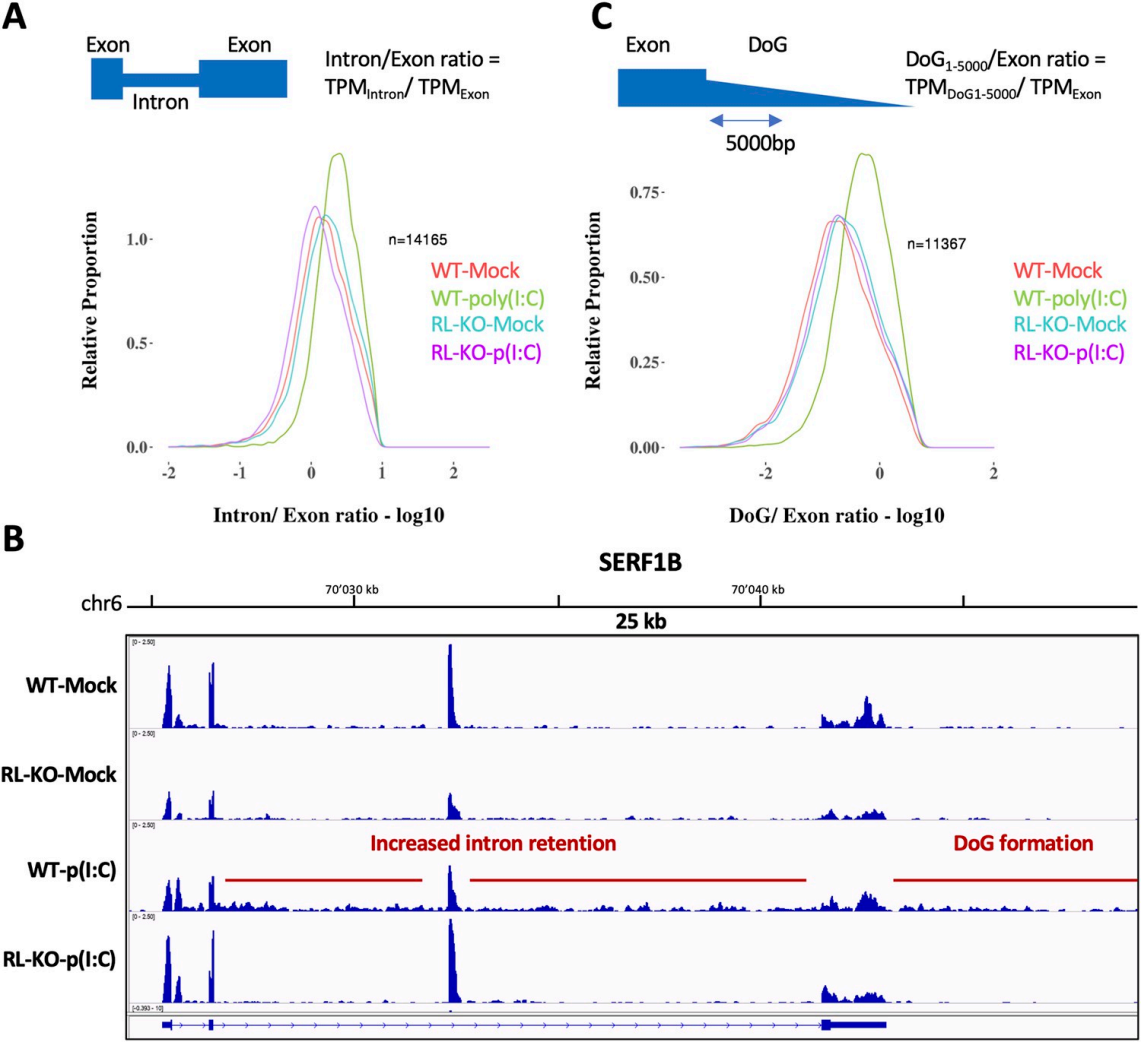
RNase L activation results in alterations to host RNA processing. (A) Distribution of intron/exon ratios of host RNAs in WT and RL-KO cells following mock or poly(I:C) lipofection. (B) IGV traces mapping to an example gene. Intron retention and DoG formation is highlighted in WT cells following poly(I:C) lipofection. (C) Distribution of DoG_1-5000bp_/exon ratios of host RNAs in WT and RL-KO cells following mock or poly(I:C) lipofection.

A striking result was that the density plots of all analyzed RNAs showed a shift to higher intron/exon ratios upon poly(I:C) treatment in WT cells, highlighting an RNase L-dependent increase in intron retention (Fig 5A). The same shift was also observed for transcripts that are either unchanged (S2 Fig), downregulated (S2 Fig) or upregulated (S3 Fig) in WT cells upon poly(I:C) treatment. Consistent with these analyses, IGV traces of multiple example genes displayed increased reads mapping to introns (Figs 5B and S3). We note that for upregulated genes, a shift towards higher intron/exon ratio is visible in wild-type cells compared to RL-KO cells, consistent with an increase in intron retention. However, an additional shift is also visible in both unstressed conditions (S3 Fig). This is caused by multiple factors that we believe are unrelated to RNase L (see methods section). These data argue that RNase L activation also decreases the efficiency of intron removal, which is supported by smFISH data of selected targets (see below).

We also observed that RNase L activation perturbs transcription termination. Specifically, we examined whether RNase L activation resulted in downstream of gene (DoG) transcriptional read-through, which are observed during viral infection and various other stresses and are consistent with defects in transcription termination [36–39]. To do this, we calculated an DoG_1-5000_/exon ratio, where DoG_1-5000_ corresponds to TPM normalized counts over the first 5000 nt after the annotated gene end (Figs 5C and S4) (see methods).

In WT cells transfected with poly(I:C), we observed DoG transcripts, which were notably absent in poly(I:C)-transfected RL-KO cells (Fig 5C). For example, IGV traces for multiple RNAs demonstrates increased reads mapping to the DoG region in WT cells treated with poly(I:C) that are absent in mock-treated WT or RL-KO cells as well as poly(I:C)-treated RL-KO cells (Figs 5B and S4).

We considered the possibility that the RNase L-dependent effects on splicing and DoG transcription identified via our RNA-seq analysis could be a technical artifact of cytoplasmic-specific RNA decay, which we postulated could increase nuclear RNA processing intermediates relative to mature, cytoplasmic RNAs. However, three observations indicate that this does not solely account for the increased intron and DoG RNA reads.

First, we did not observe RNase L-dependent RNA processing alterations in all genes, indicating specificity to RNase L-dependent RNA processing effects. For example, although both GAPDH and ACTB mRNAs are substantially reduced in the cytoplasm but not in the nucleus in an RNase L-dependent manner (S4 Fig), we did not observe increased reads mapping to the introns of GAPDH or ACTB (S5 Fig). Second, we observed increased reads mapping to specific regions of genes. For example, although we did not observe increased reads mapping to the introns of ACTB, we did observe increased reads mapping to the DoG region of ACTB (S5B Fig), indicating an RNase L-dependent increase in reads mapping to ACTB specifically in the DoG region.

Third, we confirmed RNase L-dependent DoG formation in the long non-coding RNA, NORAD, via smFISH. We stained cells with smFISH probes targeting either the NORAD RNA region or the region of the NORAD gene downstream of the transcription termination/polyadenylation site (PAS) twelve hours post-poly(I:C) lipofection (S6 Fig). Consistent with earlier results, we observed RNase L-dependent degradation of the cytosolic NORAD RNA [13], and the accumulation of NORAD RNA in the nucleus due to a block to nuclear export [20]. Importantly, we observed accumulation of NORAD RNAs with 3’ DoGs as detected by hybridization to the probe downstream of the NORAD coding region in WT (S6 Fig). DoG RNAs co-localized with NORAD RNA at both the presumable transcription sites (TS; 2 large/intense foci), as was originally described for DoGs upon various stresses [37], as well as dispersed in the nucleus as individual RNAs (S6 Fig). We did not observe DoG RNAs in RL-KO cells (S6 Fig), indicating DoG RNA inclusion on the NORAD RNA is RNase L-dependent.

These observations indicate that RNase L activation results in widespread nuclear RNA processing alterations, including changes in alternative splicing, intron retention and reduced transcription termination.

### RNase L activation alters antiviral mRNA biogenesis

**Fig 6:**
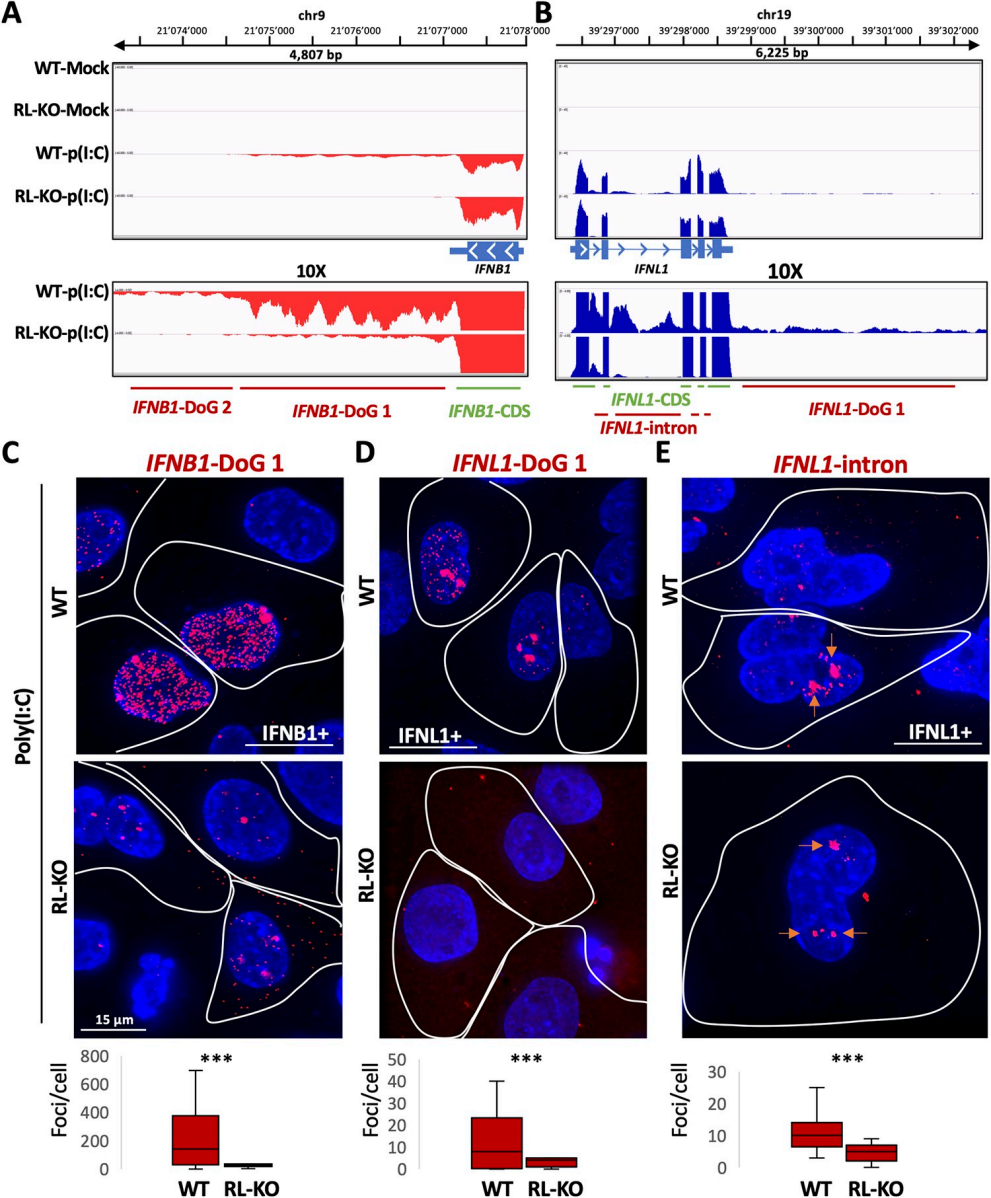
RNase L promotes DoG transcriptional read-through and intron retention in type I and type III interferon RNAs. (A) IGV traces mapping to IFNB1. Below shows the regions targeted by smFISH probes. (B) IGV traces mapping to IFNL1. Below shows the regions targeted by smFISH probes. (C) smFISH for IFNB1-DoG sixteen hours post-lipofection of poly(I:C) in WT and RL-KO cells. The cells that induced IFNB1, as determined by smFISH for the CDS of IFNB1 (S4A and S4B Fig), are demarcated by a white line. IFNB1 DoG smFISH foci are quantified in WT an RL-KO cells in the graph below. Greater than seventeen IFNB-positive cells were analyzed from at least four fields of view. (D) similar to (C) but for (D) IFNL1-DoG-1 RNA sixteen hours post-lipofection of poly(I:C). Greater than seventeen IFNB-positive cells were analyzed from at least five fields of view. (E) Similar to (D) but for IFNL1-intron RNA twelve hours post-lipofection of poly(I:C). Orange arrows indicate transcripts at the putative genomic loci of IFNL1. Greater than eleven IFNB1-positive cells were counted from at least 5 fields of view. Staining and quantification of IFNL1 CDS is shown in S4C–S4E Fig. Statistical significance was determined using student’s t-test. *< 0.05, ** is p < 0.01, *** is p < 0.001.

A key aspect of the cellular response to dsRNA is the induction of antiviral gene expression. Given this, we examined how RNase L activity affected the processing of mRNAs transcriptionally induced by dsRNA, including type I and type III interferons (IFNs). We observed that RNase L altered the RNA processing of the pre-mRNAs for both types of interferons. For example, we observed increased RNA-seq reads mapping downstream of *IFNB1* and *IFNL1* in WT cells in comparison to RL-KO cells (Fig 6A and 6B), indicating DoG RNA generation when RNase L is activated. Moreover, we observed increased reads mapping to the introns of *IFNL1*, indicative of intron retention (Fig 6B). To validate these data, we performed smFISH using probes that target the DoG of *IFNB1* and *IFNL1* or the introns of *IFNL1*. We assayed cells between 12–16 hours post-poly(I:C) to allow for more time for cells to induce interferon genes and increase the potential RNase L-mediated effects on interferon mRNAs.

Consistent with our RNA-seq analyses, several observations indicate that RNase L promotes transcriptional read-through of *IFNL1* and *IFNB1* genes. First, in a portion of WT cells that induced *IFNB1* or *IFNL1* expression in response to poly(I:C), as determined by co-smFISH for the CDS regions of these genes (S7 Fig), we observed abundant and disseminated DoG-1 smFISH foci (Fig 6C and 6D). We note that in WT cells, we did not observe abundant smFISH foci targeting the *IFNB1* DoG-2 region (S8 Fig), which is further downstream of the DoG-1 region of *IFNB1*. This correlates with lower reads mapping to this region and consistent with lower abundance of these transcripts as assessed by RNA-seq. We did observe staining for the DoG-2 region of *IFNL1*, which was predominantly localized to the presumed sites of transcription (two intense foci that stain for both the CDS and DoG) (S8 Fig). We note that we did not observe *IFN*-CDS nor *IFN*-DoG RNAs in mock-treated cells via smFISH, which is expected since interferon mRNAs are not expressed under mock conditions in A549 cells [13].

*IFNB1* and *IFNL1* DoG-1 RNA staining was significantly less abundant in RL-KO cells in comparison to WT cells (Fig 6C and 6D), despite RL-KO cells containing higher levels of CDS staining for both *IFNB1* and *IFNL1* (S7 Fig). Notably, *IFNB1*-DoG staining was mostly confined to large foci that are consistent with IFNB1 sites of transcription in RL-KO cell (Fig 6C) [13], indicating a partial hybridization of these probes to the polymerase pausing site (which is also downstream of the PAS site but upstream of the DoG). The *IFNL1* DoG-1 RNA was not observed in RL-KO cells (Fig 6D), highlighting an efficient transcription termination of the IFNL1 gene in RL-KO cells. Importantly, rescue of RNase L but not RNase L-R667A (catalytic mutant) in RL-KO cells restored *IFNB1* DoG-1 RNA generation (S9 Fig), demonstrating that the formation of DoG RNAs is dependent on RNase L catalytic activity. These data demonstrate that that RNase L promotes DoG read-through transcription of *IFNB1* and *IFNL1* genes.

*IFNL1* intron staining was largely observed at sites consistent with the IFNL1 sites of transcription (~2 intense/large foci), and this was observed in both WT and RNase L-KO cells (Fig 6E). Nevertheless, we observed increased IFNL1-intron smFISH foci in WT cells in comparison to RL-KO cells (Fig 6E). This observation is consistent with the RNA-seq data and demonstrates that RNase L activation promotes intron retention in *IFNL* transcripts.

### Alterations in RNA processing contribute to nuclear retention of antiviral mRNAs

We have previously documented that RNase L activation can lead to nuclear retention of host and viral mRNAs [20]. The observation that *IFNB1* and *IFNL1* DoG RNAs are largely localized to the nucleus led us to examine whether DoG RNA included on the *IFNB1* or *IFNL1* transcripts could contribute to their nuclear retention. To examine this, we analyzed the localization of DoG RNA and CDS RNA in WT and RL-KO cells following poly(I:C) lipofection.

**Fig 7:**
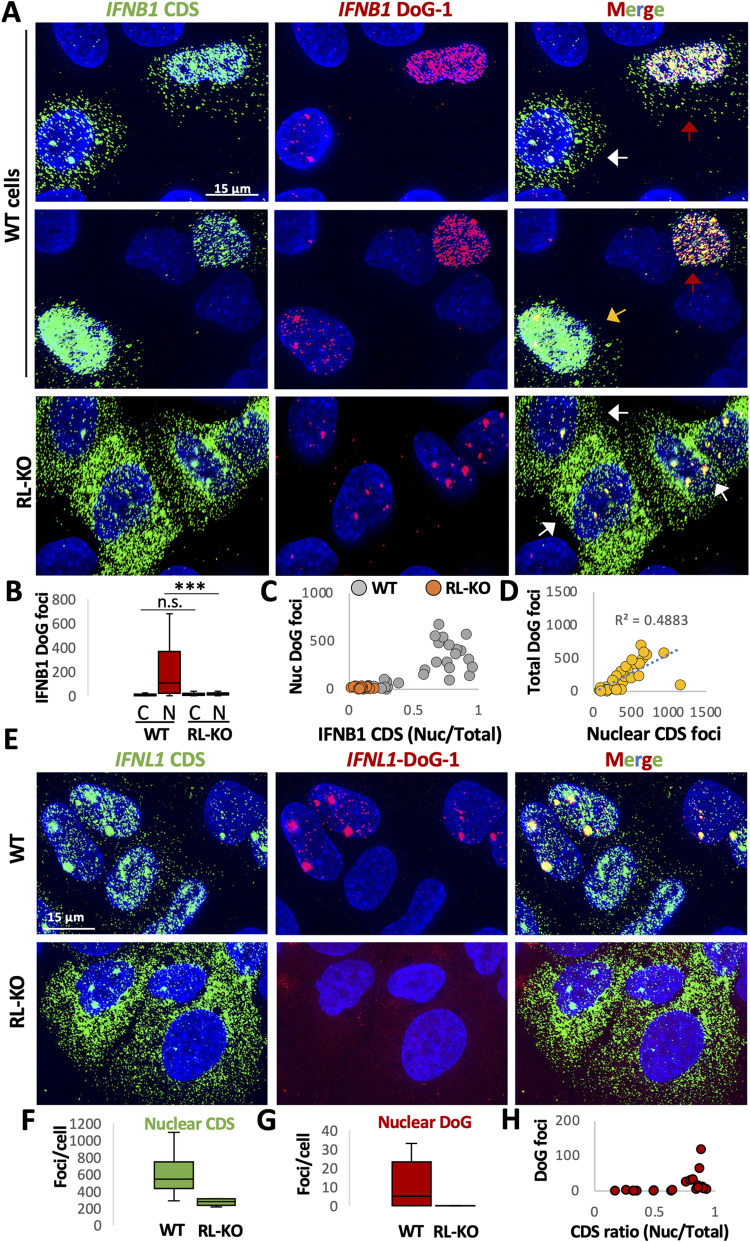
DoG RNA included on interferon-encoding mRNAs correlates with their nuclear retention. (A) Co-smFISH for the CDS and DoG-1 regions of IFNB1 sixteen hours post-lipofection of poly(I:C). White arrows indicate cells that lack both the mRNA export block and abundant DoG RNA outside the site of transcription. Red arrows indicate cells that display the mRNA export block and contain abundant and disseminated DoG RNA. Yellow arrow indicates cells that display the export block but do not contain abundant DoG RNA. (B) Box plots displaying of the number of IFNB1-DoG foci localized to the nucleus (n) or cytoplasm (c) in WT or RL-KO cells as represented in (A). Greater than seventeen IFNB-positive cells were analyzed from at least four fields of view. (C) Scatter plot of the ratio (nucleus/cytoplasm) of IFNB1-CDS foci (x-axis) and nuclear IFNB1-DoG foci (y-axis) show positive correlation between DoG RNA and nuclear retention. (D) Scatter plots of the quantity of nuclear IFNB1-DoG foci (y-axis) and the quantity of nuclear IFNB1-CDS foci shows IFNB1 DoG RNA increase as the absolute number of IFNB1 CDS smFISH in the nucleus increases. (E) Co-smFISH for the CDS and DoG-1 regions of IFNL1 sixteen hours post-lipofection of poly(I:C). (F and G) Quantification of (F) nuclear IFNL1-DoG-1 foci or (G) IFNL1-CDS foci as represented in (E). Greater than seventeen IFNB-positive cells were analyzed from at least five fields of view. (H) Scatterplot of the ratio (nucleus/cytoplasm) of IFNL1-CDS foci (x-axis) and nuclear IFNL1-DoG foci (y-axis). Statistical significance was determined using student’s t-test. *< 0.05, ** is p < 0.01, *** is p < 0.001.

Several observations indicate that DoG production from the IFNB1 mRNA contributes to its nuclear retention. First, the *IFNB1*-DoG RNAs are almost exclusively localized in the nucleus, even in cells that have exported *IFNB*1 mRNAs that only stain for the CDS region to the cytosol (Fig 7A and 7B; red arrows). Second, in WT cells with abundant and disseminated IFNB1-DoG foci, the DoG foci co-localize with IFNB1-CDS foci that are retained in the nucleus (Fig 7A; red arrows). Third, *IFNB1*-CDS foci located in the cytoplasm only contain DoGs in very rare cases (Fig 7A; red arrows). Lastly, we observed that the abundance of DoG foci positively correlates with the ratio (nuclear/total) of *IFNB1*-CDS or the absolute number of nuclear *IFNB1*-CDS foci (Fig 7C and 7D). We observed similar effects at earlier times (eight hours) post-transfection with poly(I:C) (S10 Fig). Although we cannot rule out the possibility that the DoG RNA sensitizes the *IFNB1* transcript to RNase L-mediated degradation in the cytoplasm, these data argue that IFNB1 RNA transcripts containing the DoG RNA are not exported to the cytoplasm,

However, our data suggest that additional mechanisms can inhibit mRNA export. First, we observed cells in which most nuclear-retained *IFNB1*-CDS RNAs did not contain *IFNB1*-DoG RNA (Fig 7A, yellow arrow). The accumulation of *IFNB1* mRNAs in the nucleus that do not hybridize to DoG probes suggests two possibilities. First, there could be a mechanism independent of DoG transcriptional read-through that inhibits *IFNB1* mRNA export, which is supported by our analysis of *IFNL1* mRNA (see below). However, we cannot rule out the formal possibility that all the nuclear retained *IFNB1* RNAs could contain DoGs, but with some being too short to hybridize to the DoG probes. However, we did not observe a gradual reduction in RNA-seq reads in the DoG-1 region of *IFNB1* mRNAs (Fig 6A). Therefore, we consider this latter possibility unlikely.

The examination of *IFNL1* mRNAs provides additional evidence for a block to mRNA export that is independent of RNA processing defects. Specifically, we observed that most of the nuclear-retained *IFNL1* mRNA did not hybridize to smFISH probes targeting *IFNL1* introns (S7 Fig) or DoG-1 RNA (Fig 7E). Moreover, the number of nuclear-retained *IFNL1* transcripts (CDS) was much higher than the number of both DoG-1 and intron foci in the nucleus (Fig 7F and 7G).

Nevertheless, *IFNL1* mRNAs that contained DoGs or intron sequences were mostly nuclear-retained (Figs 7E and S7), and abundant *IFNL1* DoG-1 RNA production was only observed in cells displaying nuclear retention of *IFNL1* CDS transcripts (Fig 7H). These data are consistent with defects in RNA processing limiting RNA export. Taken together, these observations demonstrate the defects in RNA processing can contribute to nuclear retention of mRNAs after RNase L activation, but also provide evidence for a block to mRNA export independent of DoG transcription and intron retention.

### RNase L-dependent re-localization of RBPs and RNA processing alterations occur during viral infection

One limitation of the above experiments is that we have used poly(I:C) as a dsRNA mimic of viral infection. To verify that these alterations were relevant to viral infection, we examined whether RNase L-dependent re-localization of RBPs and nuclear RNA processing alterations occurred during viral infection. We analyzed PABPC1 localization via immunofluorescence in WT and RL-KO A549 cells following infection with either dengue virus serotype 2 (DENV2) or SARS-CoV-2 (using A549^ACE2^ cells), both of which activate RNase L [4,19,20]. To identify infected cells, we co-stained the viral mRNAs via single-molecule fluorescence in situ hybridization (smFISH), while alterations in RNA processing were examined by smFISH for *IFNB1* of *IFNL1* DoGs or introns.

**Fig 8:**
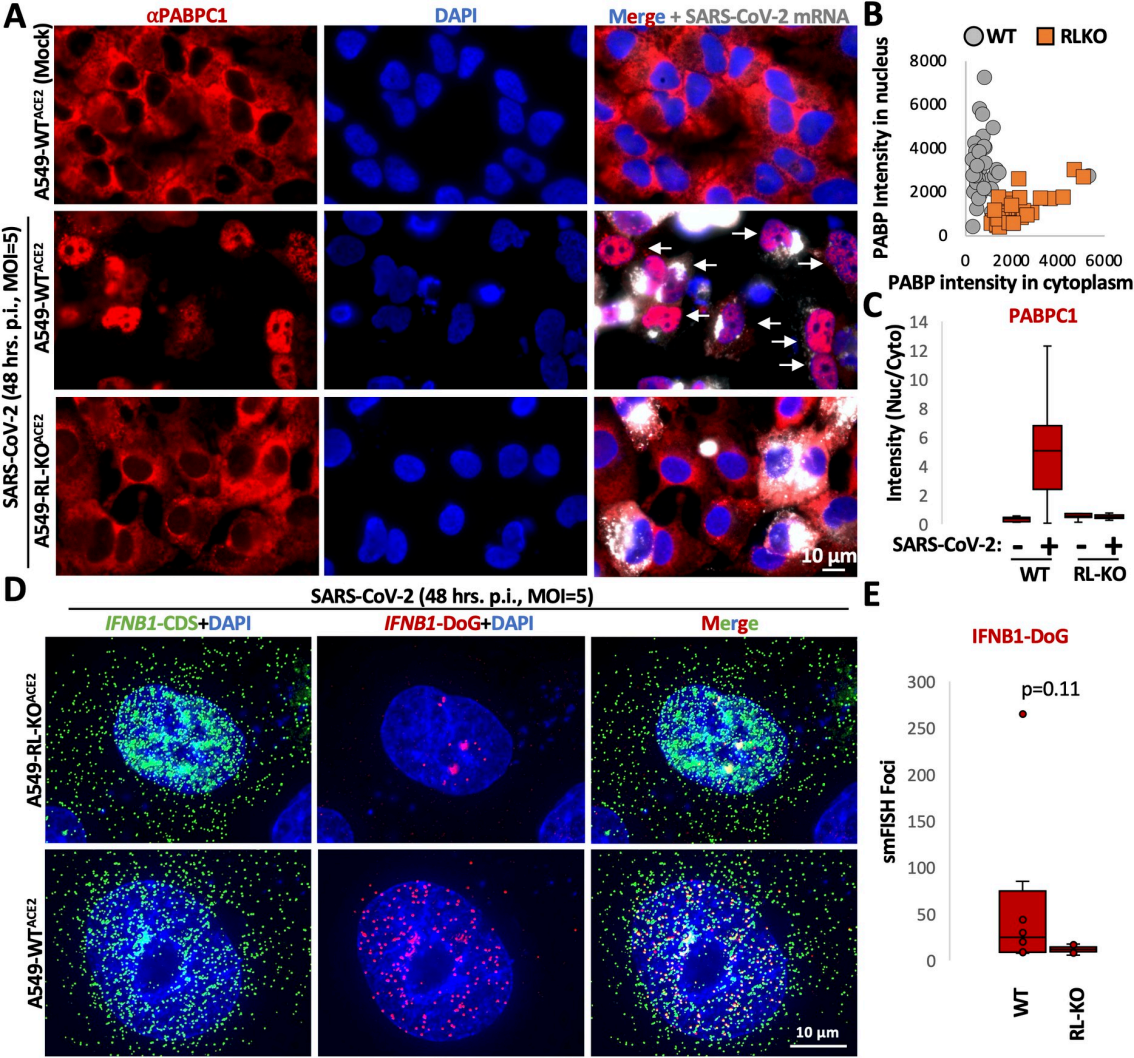
RNase L promotes nuclear RBP influx and DoG transcriptional read-through of IFNB1 during SARS-CoV-2 infection. (A) Immunofluorescence assay for PABP in WT^ACE2^ and RL-KO^ACE2^ A549 cells forty-eight hours post-infection with SARS-CoV-2 (MOI = 5) or mock-infected WT cells. To identify infected cells, smFISH for SARS-CoV-2 ORF1b mRNA was performed. (B) Scatter plot of mean intensity values for PABP staining in the nucleus (y-axis) or cytoplasm (x-axis) in mock or SARS-CoV-2-infected WT^ACE2^ or RL-KO^ACE2^ A549 cells as represented in (A). Dots represent individual cells. Between 20–44 cells were analyzed for group from at least three fields of view. (C) Box plot of the ratio (nucleus/cytoplasm) of the mean intensity of PABP as represented in (A). (D) smFISH for IFNB1-CDS and IFNB1-DoG forty-eight hours post-infection with SARS-CoV-2 in WT^ACE2^ or RL-KO^ACE2^ A549 cells (MOI = 5). (E) Quantification of IFNB1 DoG RNA in WT and RL-KO cells. Statistical significance was determined using student’s t-test.

These analyses showed that RNase L activation promotes PABPC1 translocation to the nucleus and alteration of RNA processing in response to SARS-CoV-2 infection (Fig 8E). Specifically, most WT A549 cells infected with SARS-CoV-2, as detected by smFISH for the viral RNA, displayed accumulation of PABPC1 in the nucleus by 48 hours post-infection (Fig 8A–8C; white arrows), which was not observed in RL-KO cells.

Importantly, smFISH for *IFNB1* CDS and DoG-1 showed the production of *IFNB1* DoGs during SARS-CoV-2 infection in both WT and RL-KO cells (Fig 8D). Similar to our results with poly(I:C), *IFNB1*-DoG RNAs were largely localized to the presumed site of transcription in RL-KO cells, whereas we observed abundant and dispersed *IFNB1* DoG RNA in the nucleus of WT cells (Fig 8D). Notably, *IFNB1* transcripts in WT cells that contained DoG RNAs were largely localized in the nucleus (Fig 8D), thus indicating that the DoG RNA reduces the ability of *IFNB1* transcript to accumulate in the cytosol. We did not observe RL-KO cells that generated abundant IFNB1 DoG RNA as observed in WT cells (Fig 8E). Nevertheless, we observe nuclear retention of *IFNB1*-CDS in RL-KO cells as previously described [19], and we suggest that this occurs because the SARS-CoV-2 Nsp1 protein is sufficient to inhibit mRNA export via degradation the bulk of cytosolic mRNA independently of RNase L and inhibiting RNA export factors [19,40].

**Fig 9:**
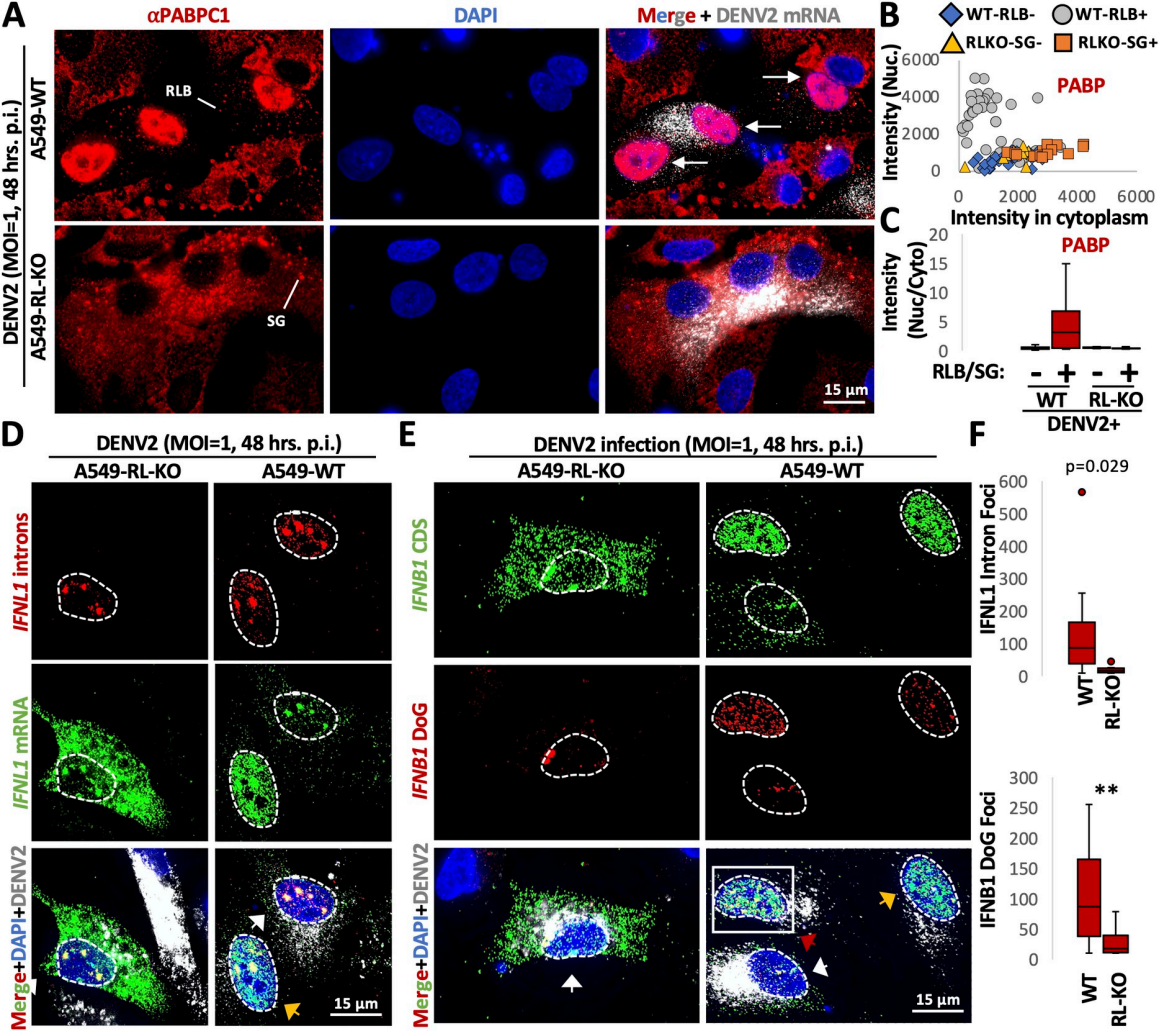
RNase L promotes nuclear RBP influx and DoG transcriptional read-through and intron retention during dengue virus infection. (A) Immunofluorescence assay for PABP in WT and RL-KO A549 cells infected with dengue virus serotype 2 (DENV2) forty-eight hours post-infection (MOI = 1). smFISH for DENV2 mRNA was performed to identify infected cells. (B) Scatter plot of mean intensity values for PABP staining in WT or RL-KO cells that did or did not activate the dsRNA response based on RLB assembly (WT cells) or SG assembly (RL-KO) cells as represented in (A). (C) Box plot of the ratio (nucleus/cytoplasm) of the mean intensity of PABP (B). (D) smFISH for IFNL1-CDS, IFNL1-intron, and DENV mRNA in WT or RL-KO cells forty-eight hours post-infection with DENV2 (MOI = 1). (E) smFISH for IFNB1-CDS and IFNB1-DoG in WT or RL-KO cells forty-eight hours post-infection with DENV2 (MOI = 1). (F) Box plots quantifying the smFISH from individual cells (D and E). Twenty-five WT cells and eight RL-KO cells were analyzed from greater than five fields of view for IFNB-DoG-1. Fifteen WT cells and nine RL-KO cells were analyzed from greater than five fields of view. Statistical significance was determined using student’s t-test. *< 0.05, ** is p < 0.01, *** is p < 0.001.

Similar results were observed with DENV-infected cells, although approximately half of DENV-infected cells do not activate RNase L-mediated mRNA decay [20] (S11 Fig). Thus, approximately half of DENV-infected cells did not display translocation of PABP to the nucleus as expected (Figs 9A and S11). However, we observed that many DENV2-infected WT cells assembled RLBs, whereas many DENV2-infected RL-KO cells assembled SGs (Fig 9A). This allowed us to identify cells that activated the dsRNA immune response. We then calculated the nuclear to cytoplasmic PABP in DENV-infected WT cells that activated RNase L (RLB+) or RL-KO cells that activated PKR (SG+) in comparison to cells that did not activate dsRNA response (RL- for WT cells or SG- for RL-KO cells). These analyses revealed a substantial increase in nuclear PABPC1 specifically in DENV2-infected WT cells that activated RNase L (Fig 9B and 9C).

Examination of RNA processing defects in DENV2-infected cells via smFISH revealed that RNase L-dependent RNA processing alterations occurred during dengue virus infection. Specifically, we observed that *IFNL1*-intron and IFNB1-DoG were both higher in DENV2-infected WT cells comparison to RL-KO cells (Fig 9D–9F).

Taken together, the analysis of *IFN* mRNAs documents that RNase L activation either due to poly(I:C) transfection or viral infection triggers the accumulation of RBPs in the nucleus and affects nuclear RNA processing of antiviral mRNAs, with a stronger effect on transcriptional termination leading to the production of DoGs for these mRNAs.

### Nuclear PABP accumulation is not required for mRNA export inhibition or DoG transcriptional read-through of IFNB1

An unresolved issue is the specific RBPs that accumulate in the nucleus and which transcripts are affected by one or more specific RBPs. One hypothesis is that PABPC1 accumulation in the nucleus could alter *IFNB1* RNA export and processing. This is based on earlier work that the accumulation of PABPC1 in the nucleus inhibits mRNA export, promotes hyperadenylation of mRNAs, and inhibits transcription [23,25,26]. Therefore, we asked if PABPC1 translocation to the nucleus was responsible for the nuclear export of *IFNB1* mRNA and DoG transcriptional read-through of the *IFNB1* gene.

**Fig 10:**
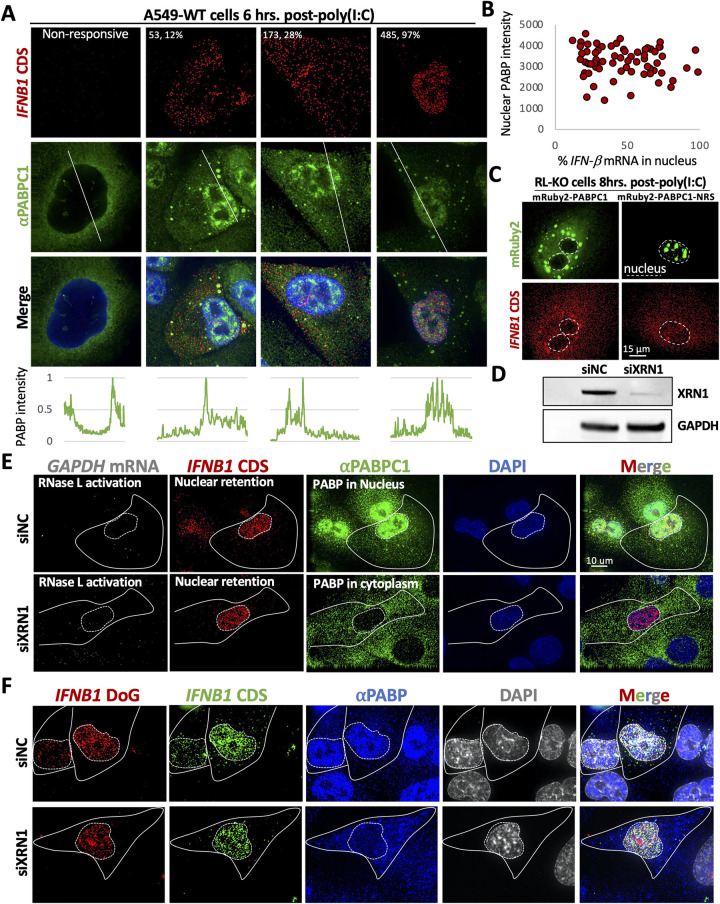
PABP accumulation in the nucleus is not sufficient for mRNA export block or DoG transcriptional read-through of IFNB1. (A) Immunofluorescence for PABPC1 and smFISH for IFNB1 mRNA in A549 six hours after poly(I:C) transfection. Left panel shows a non-responsive cell that did not activate RNase L or induce IFNB1. The numbers in the top left corner are the number of nuclear IFNB smFISH foci and the percentage that those represent out of the total smFISH foci in the cell. Below the panels are intensity plots of PABPC1 signal as indicated by the white line. (B) Scatter plot of nuclear PABP intensity (y-axis) and the percent of IFNB1 mRNA in the nucleus (x-axis). (C) smFISH for IFNB1 mRNA in A549-RL-KO cells that express either mRuby2-PABPC1 or mRuby2-PABPC1 containing a nuclear retention signal (NRS). Quantification the percent of IFNB1 mRNA in the nucleus is shown in S12A Fig. (D) Western blot of XRN1 from A549 transfected with XRN1-targeting siRNAs (siXRN1) or negative control siRNAs (siNC). (E) IF for PABP and smFISH for GAPDH mRNA and IFNB1 mRNA in A549-WT cells transfected with either siNC or siXRN1. (F) IF for PABP and smFISH for IFNB1 CDS and DoG-1 in A549-WT cells transfected with either siNC or siXRN1. Quantification of both (E) and (F) are shown in S12B and S12C Fig.

To address this hypothesis, we first asked whether nuclear PABPC1 accumulation correlated to nuclear *IFNB1* mRNA retention by measuring the immunofluorescence intensity of PABPC1 in the nucleus of cells that induced *IFNB1* mRNA, which we measured by smFISH. These analyses revealed that *IFNB1* mRNA could be primarily localized to the cytoplasm in cells that contained nuclear PABPC1 accumulation (Fig 10A), and that the nuclear localization of PABPC1 did not correlate to *IFNB1* mRNA nuclear retention (Fig 10B).

*IFNB* mRNA could be induced prior to nuclear PABP translocation, thus negating the potential effect of PABP on *IFNB1* mRNA export. Therefore, to more directly test whether nuclear PABP could inhibit *IFNB1* mRNA export, we generated RL-KO A549 cell lines that express either mRuby2-PABPC1 or mRuby2-PABPC1 that contained a nuclear retention signal (mRuby2-PABPC1-NRS), similar to previous studies [23]. Six hours after poly(I:C) transfection, we performed smFISH for *IFNB1* mRNA. Importantly, in cells that contained nuclear mRuby2-PABPC1-NRS, *IFNB1* mRNA was abundant in the cytoplasm (Fig 10C) and equivalent to levels observed in cell expressing mRuby2-PABPC1 (S12 Fig). These data further support that nuclear PABPC1 is not the primary RBP responsible for inhibiting *IFNB1* mRNA export.

Lastly, since depletion of XRN1 inhibits the import of PABPC1 into the nucleus during mRNA degradation initiated by MHV68 muSOX protein [25], we transfected A549-WT cells with either a negative control siRNA (siNC) or siRNAs targeting the *XRN1* transcript (siXRN1). Western blot analysis demonstrated siXRN1-specific reduction of XRN1 protein (Fig 10D). Forty-eight hours post-transfection of siRNAs, we transfected cells with poly(I:C). As expected, cells transfected with siXRN1 resulted in inhibition of PABPC1 translocation to the nucleus in cells that initiated RNase L-mediated mRNA decay, as determined by the lack of *GAPDH* mRNA (Figs 10E and S12). Importantly, we observed similar nuclear retention of *IFNB1* mRNA in cells that initiated mRNA decay but did not display nuclear PABPC1 accumulation in comparison to cells that displayed nuclear PABP accumulation (Figs 10E and S12). Moreover, we observed abundant *IFNB1*-DoG RNA production in cells that did not display nuclear PABPC1 accumulation (Figs 10F and S12).

**Fig 11:**
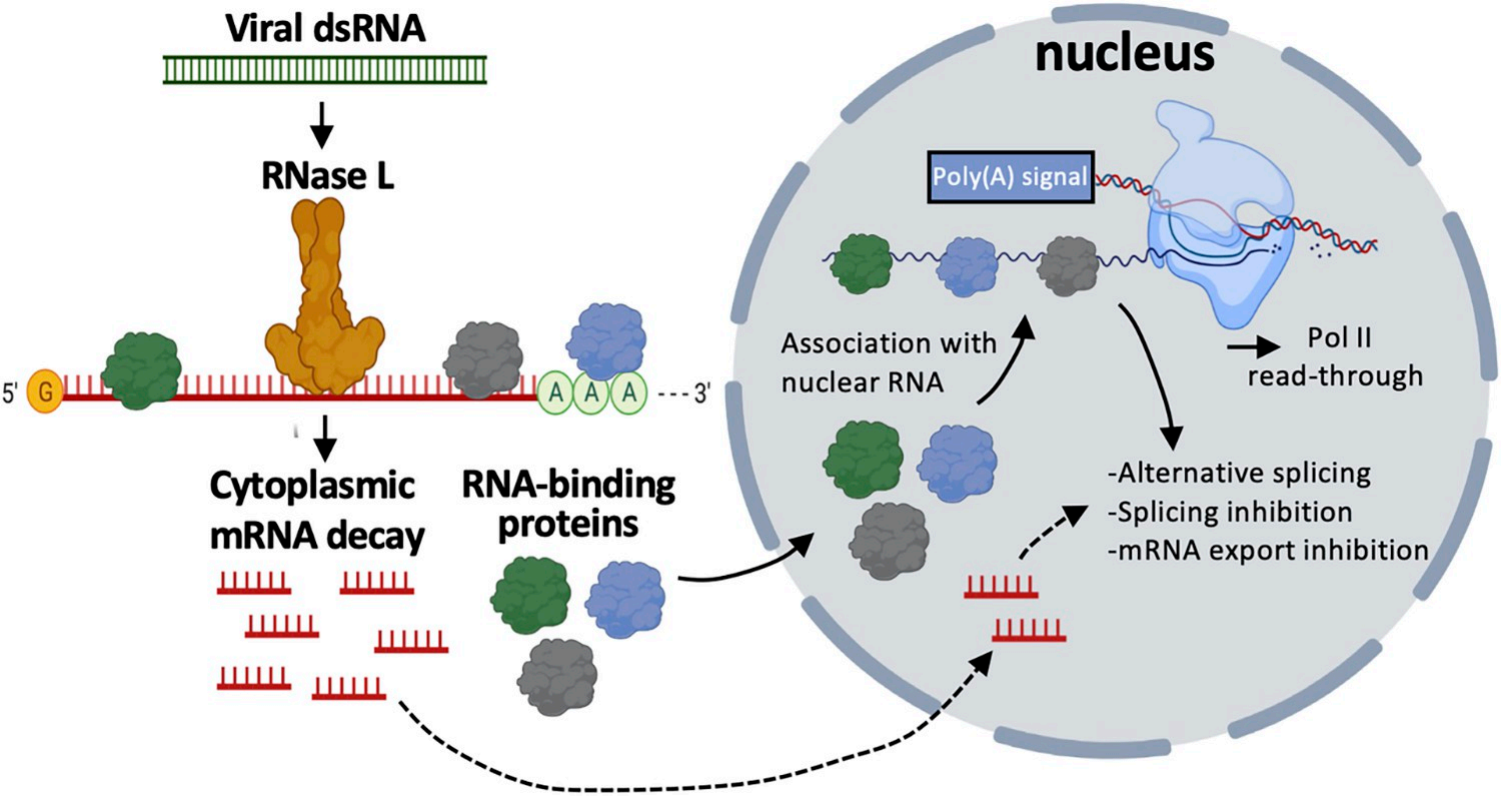
Model for RNase L-mediated regulation of nuclear RNA processing. Activation of RNase L in response to viral double-stranded RNA (dsRNA) results decay of cytoplasmic RNAs. RNA-binding proteins (RBPs) disassociate from degraded cytoplasmic RNA and shuttle to the nucleus where they bind to nuclear RNA. The binding of RBPs to nuclear RNA alters RNA processing events. In addition, degraded cytoplasmic RNA may also antagonize nuclear RNA processing.

Combined, these data suggest that nuclear PABPC1 accumulation is not the primary mechanism that inhibits IFNB1 mRNA export nor DoG transcriptional read-through of IFNB1. This indicates that a different RBP or several other RBPs in combination, as well as potentially RNase L-cleaved RNAs, in addition to PABPC1 are required for RNase L-dependent export inhibition and RNA processing alterations (Fig 11).

## Discussion

Several observations support that RNase L-mediated RNA decay results in re-localization of RBPs from the cytoplasm to the nucleus, which in turn alters nuclear RNA processing (Fig 11). First, we observed that several RBPs concentrate in the nucleus following RNase L activation (Fig 1). Second, RNase L and RNase L-mediated RNA decay are localized the cytoplasm (Fig 2A–2C), sparing nuclear RNA from degradation. Moreover, intact nuclear RNA is required for the accumulation of RBPs to the nucleus, suggesting RBPs associate with nuclear RNA upon influx into the nucleus (Fig 3). Third, our RNA-seq analyses show that RNase L activation triggers global alterations in splicing and transcription termination (Figs 4 and 5). Fourth, our smFISH analyses show that the number of DoGs and introns are increased in WT cells in comparison to RL-KO cells in response to poly(I:C) lipofection, and that these RNAs can be retained at the sites of transcription or disseminated in the nucleus (Figs 6 and 7). Importantly, we show that RNase L-dependent RBP re-localization to the nucleus and alterations to type I and type III interferon mRNA biogenesis occur during dengue virus or SARS-CoV-2 infection (Figs 8 and 9).

We suggest that multiple different RBPs may affect specific RNA processing events in an idiosyncratic manner, whereby a specific RBP regulates specific transcripts. This is based on the observation that individual RBPs can autoregulate their own RNA processing by competing for RNA processing sites, and therefore when the RBPs are in excess they reduce the production of their own mRNA [25]. This illustrates the general principle that increasing concentrations of individual RBPs only affects a specific set of transcripts due to the nature of the RNA sequence surrounding critical RNA processing signals.

This work highlights the conclusion that the availability of RNA-binding sites can influence the distribution of RBPs between the cytosol and nucleus, which explains multiple observations in the literature. First, during the integrated stress response, the release of mRNAs from ribosomes due to phosphorylation of eIF2α exposes new RNA-binding sites for RBPs in the cytoplasm, which causes nuclear RBPs to re-distribute to the cytoplasm and/or stress granules (Fig 1, HuR and TIA-1) [29]. Similarly, the export of RBPs from the nucleus in response to actinomycin D-mediated inhibition of transcription can be understood as a loss of nuclear RNA leading RBPs to associate with cytosolic RNA [41]. Finally, when cytosolic RNAs are degraded in response to proteins encoded by herpesvirus, numerous RBPs are observed to accumulate in the nucleus [25]. Thus, sub-cellular changes in RNA abundance and/or availability of RNA binding sites dictates RBP localization for many RBPs. Indeed, a number of RBPs known to act in mRNA processing are themselves differentially spliced upon exposure to poly(I:C) in an RNase L-dependent manner (Fig 4). Interestingly, some RBPs are less affected by cytosolic RNA decay with respect to their localization. For example, G3BP1 shows only a small increase in nuclear accumulation following RNase L activation (Fig 1B and 1C). We suspect that this is due to their association with protein substrates that regulate their localization, or a slower intrinsic rate of protein import into the nucleus.

RNase L-mediated regulation of RBP localization represents a new and important potential antiviral mechanism since studies have shown that host RBPs interact with and can affect viral replication [42]. Since viruses replicate specifically in either the nucleus or cytoplasm, the RNase L-mediated re-localization of RBPs between the cytoplasm and nucleus may promote the antiviral function of RBPs and/or viruses may use RNA decay to avoid host RBPs. Notably, several viruses, including Kaposi’s sarcoma-associated herpesvirus (KSHV), murine gammaherpesvirus 68 (MHV68), and herpes simplex virus 1 (HSV-1) encode for proteins (SOX, muSOX, VHS, respectively) that degrade cellular RNAs, inhibit nuclear mRNA export, and cause nuclear RNA-binding protein influx [21–26,43]. Interestingly, KSHV encodes an RNA, PAN, that rescues nuclear export of viral mRNAs [44]. An intriguing possibility is that the PAN RNA titrates RBPs that interfere with nuclear export, thereby allowing export of the herpes mRNAs. Thus, RBP re-localization can be either host- or viral-mediated and can potentially alter both host and viral gene expression. Lastly, the inhibition of host-mediated RNA decay by viruses, such as by flavivirus sfRNA-mediated inhibition of XRN-1 or poliovirus-mediated inhibition of RNase L [9,45], may regulate host RBP interactions with the viral RNA. Elucidating how overall RNA abundance and subcellular location regulates the availability RBPs to modulate both host and viral RNA function will be an important issue to address.

Notably, muSOX and SOX proteins both localize to the nucleus as well as the cytoplasm. However, their presence in the nucleus does not reduce RNA levels as assessed by FISH for poly(A)+ RNA or northern blot of reporter RNAs [21,22]. Moreover, nuclear localization of SOX/muSOX does not inhibit PABP accumulation in the nucleus [22]. These observations contrast RNase L, which is exclusively localized to the cytoplasm (Fig 2A–2C). Importantly, when we localized RNase L to the nucleus, we observed a substantial reduction in poly(A) RNA (Fig 2F and 2G). Moreover, we observed a reduction of PABPC1, PABPC4, TIA1, and HuR in the nucleus (Fig 3A–3H). These data demonstrate that the endoribonucleolytic cleavage of RNA in the nucleus by RNase L can initiate RNA decay in the nucleus, resulting in re-partitioning of RBPs back to the cytoplasm. Why muSOX and SOX do not degrade nuclear RNA and thus re-partition RBPs back to the cytoplasm is unclear. This suggests that RNase L and SOX/muSOX mechanisms for cytoplasmic RNA decay are distinct.

An unexpected observation is that SARS-CoV-2 infection, which results in rapid degradation of host mRNAs even in the absence of RNase L through the action of the SARS-CoV-2 Nsp1 protein [19], does not trigger robust PABPC1 translocation to the nucleus as demonstrated by the lack of nuclear PABP staining in SARS-CoV-2 infected RL-KO cells (Fig 8). We suggest two possibilities to explain this phenomenon. First, the difference in the RNA decay mechanisms between RNase L and SARS-CoV-2 Nsp1 could result in differential release of RBPs such as PABP from RNAs. Second, SARS-CoV-2 may prevent RBP influx into the nucleus, and RNase L overcomes this inhibitory mechanism. Nevertheless, during SARS-CoV-2 infection we still observed more *IFNB1* DoG RNAs in WT cells than RL-KO cells, indicating that DoG RNA generation is a consequence of RNase L activation in response to SARS-CoV-2 infection. Whether this is beneficial for SARS-CoV-2 since it reduces host antiviral gene expression is unclear. However, since RNase L reduces SARS-CoV-2 replication in cultured cells by only 3–4 fold, presumably via decay of the viral mRNA [4,19], perhaps the potential loss of fitness of allowing RNase L activation is outweighed by the perturbation in host interferon production *in vivo*.

Our data establish that RNase L activation promotes the formation of DoG transcription read-through. Interestingly, while DoG transcripts are generally retained at the site of transcription [36], we observed abundant *IFNB1* transcripts that contained DoG RNA disseminated throughout the nucleus (Figs 6 and 7). Moreover, the *IFNB1* transcripts with DoG RNA were almost exclusively localized to the nucleus, whereas IFNB1 transcripts in the same cells without the DoG RNA were localized to the cytoplasm. These observations suggest that the DoG RNA on *IFNB1* transcripts might inhibit their mRNA export, even after their release from the site of transcription. Alternatively, the DoG RNA may sensitize *IFNB1* transcripts to RNase L in the cytoplasm. Regardless, the inclusion of downstream elements on the *IFNB1* mRNA transcript may present a new RNase L-dependent regulatory mechanism. Lastly, since the inhibition of mRNA export via RNase L activation reduces interferon proteins synthesis [20] and DoG RNA correlates with the export block (Fig 7), we suggest that the production of DoG RNAs due to activation of RNase L interferes with interferon production. Future work will aim to directly test the functions of DoG RNA on *IFNB1* mRNA biogenesis, stability, and translation.

The RNA processing defects promoted by RNase L activation, such as DoG read-through transcription and splicing alterations (alternative splicing and intron retention), may prove to be new antiviral mechanisms that perturb viral gene expression or promote the host immune response. Moreover, since RBPs can directly modulate transcription [21,46], this work strongly implies that widespread cytoplasmic RNA degradation by RNase L will lead to changes in transcription of multiple genes due to the influx of RBPs into the nucleus. Future work will examine these potential functions and address the specific mechanism by which RNase L activation alters nuclear RNA processing and transcription.

## Materials & methods

### Cell culture

Parental and RNase L knockout A549 cell lines are described in [13]. Cells were maintained at 5% CO_2_ and 37 degrees Celsius in Dulbecco’s modified eagle’ medium (DMEM) supplemented with fetal bovine serum (FBS; 10% v/v) and penicillin/streptomycin (1% v/v). Routine testing for mycoplasma contamination was performed by the cell culture core facility. African green monkey kidney cells (Vero E6, ATCC CRL-1586) were maintained at 5% CO_2_ and 37 degrees Celsius in DMEM supplemented with FBS (10% v/v), 2 mM non-essential amino acids, 2 mM l-glutamine, and 25 mM HEPES buffer.

For transfections, high-molecular weight poly(I:C) (InvivoGen: tlrl-pic) and 3-μl of lipofectamine 2000 (Thermo Fisher Scientific) per 1-ug or poly(I:C) was used per manufacturer’s instructions. Cells were transfected with 500-ng of poly(I:C) per volume of media (1-ug in 6-well format).

### Viral infections

Viral infections are described in [19,20]. Briefly, cells were infected with DENV serotype 2 16681 strain at MOI of 1.0. Cells were fixed 48 hours after infection. SARS-CoV-2/WA/20/01 (GenBank MT020880; BEI Resources: NR-52881) was passaged in Vero E6 cells, and viral titer was determined via plaque assay on Vero E6 as previously described in [47]. A multiplicity of infection (MOI) of 5 was used. All SARS-CoV-2 infections were conducted under biosafety level 3 conditions at Colorado State University. Dengue virus infections were conducted under biosafety level 3 conditions at University of Colorado, Boulder. For infections, cells were seeded in 6-wells format onto cover slips and inoculated twenty-four hours later. Cells were fixed in 4% paraformaldehyde and phosphate-buffered saline (PBS) for 20 minutes, followed by three five-minute washes with 1X PBS, and stored in 75% ethanol.

### Plasmids

The generation of the pLenti-ef1-blast–mRuby-2-PABPC1 lentiviral plasmid is described in [15]. Briefly, the mRuby-2 coding sequence was amplified via PCR, the PABPC1 coding sequence was amplified via PCR from the pCI–MS2V5–PABPC1 (Addgene catalog no. 65807), and the sequences were fused with and inserted into the XhoI/XbaI sites of pLenti–EF1–BLAST vector using in-fusion (S1 Data). To generate the pLenti–mRuby-2–PABPC1-NRS, the hnRNPC1-derived nuclear retention signal [23] was inserted on the c-terminus of the PABPC1 ORF via PCR and subsequent in-fusion insertion into pLenti-ef1-blast (S1 Data).

### RNA-seq analysis

Our previously published RNA-seq data [13] were re-processed using a Nextflow pipeline (https://github.com/Dowell-Lab/RNAseq-Flow) as follows. Reads were trimmed using BBduk version 38.05 with the following flags ktrim = r qtrim = r, trimq = 10, k = 23 mink = 11 hdist = 1 maq = 10 minlen = 25 (Institute, J. G. Bbmap. https://sourceforge.net/projects/bbmap/ (2015)). Trimmed reads were mapped to the human reference genome (GRCh38/hg38) using HISAT2 version 2.1.0 with the very-sensitive, pen-noncansplice 14, mp 1,0, and sp 3,1 flags [48]. SAMtools version 1.8 was used to convert sam files to sorted bam files [49]. TDV files were generated using IGV-tools 2.3.75 [50]. Normalized TDF files that were generated by the pipeline were used to visualize representative gene traces with the Integrative Genomics Viewer [50]. Exon or intron reads were counted for each sorted BAM file over annotated genes in the RefSeq hg38 genome using featureCounts (Rsubread/2.0.1, [51]. GTFtools was used to determine intron boundaries [52] and to generate an introns only gtf file. RNA isoforms were filtered for the highest expressed isoform equivalent to the highest FPKM value in the WT condition. DESeq2/1.26.0 was used for differential expression analysis. ERCC RNA Spike-Ins were used to estimate and correct size factors in DESeq2. In particular, ERCC Spike-Ins reads were mapped using the same Nextflow pipeline to the ERCC sequences (https://tools.thermofisher.com/content/sfs/manuals/ERCC92.zip), counted using featureCounts and the ERCC Spike-Ins size factors derived by DESeq2. Significant differentially upregulated or downregulated RNAs were selected by baseMean >5, padj < 0.05 and a log2fold change of 1. Nonsignificant genes were selected by padj > 0.05 and log2fold change of -0.5 to 0.5.

Intron retention analysis was performed on all RNAs with the Deseq2 baseMean cutoff > 30, to remove RNAs with too low counts. Intron/exon ratios were derived by diving the TPM normalized intron counts by TPM normalized exon counts. Intron/exon ratios > 8.5 were a result from “towers” in intronic regions, counts in non-annotated regions, noise due to low counts or read-through from an upstream gene. Therefore, all intron/exon ratios >8.5 were removed from analysis.

We note that for upregulated genes, a shift towards higher intron/exon ratio is visible in wild-type cells compared to RL-KO cells, consistent with an increase in intron retention. However, an additional shift is also visible in both unstressed conditions (S2E Fig). This is caused by multiple factors. First, a few genes showed high intron retention in RL-KO cells even without stress. Second, transcriptional read-through from upstream genes (see below), causes increased ratios. The majority of such transcripts is filtered out by initial filtering steps (material and methods), however, especially for upregulated genes, some transcripts remain and can cause false positive increased ratios. Third, wrong gene annotations (e.g., an exon with increased reads is counted within intronic region), can make increase in ratio for upregulated genes more pronounced. And lastly, despite an overall increase in IR in WT cells upon poly(I:C) (S2E Fig), the intron/exon ratio decreases due to the increased expression and higher exon counts over the intron counts, especially for genes with short introns and large exons.

DoG formation was estimated similarly, by generating TPM normalized counts over the first 5000 pb following the exon annotation. These TPM normalized DoG_1-5000_ counts were divided by the TPM normalized exon counts to derive and a DoG/exon ratio. For the same reasons as describe for intron/exon ratios, DoG/exon ratios > 5 were removed from analysis. Moreover, small RNAs (exon size < 300bp) were removed from analysis.

In our ratio analysis, genes were not filtered for “clean” genes, genes that have counts due to expression and not due to read-through transcription from up-coming gene [53]. However, many genes that were not expression but showed read-through transcription from up-coming gene were filtered out by the intron/exon ratios >15 and DoG/exon ratios > 5 step. We do anticipate false-positives left in our analysis, especially in the upregulated group. Figures were made using ggplot2/3.3.3.

For the splicing analysis, raw fastq reads were trimmed to remove adapters and low quality reads with bbduk/38.79 [54], aligned to the hg38 genome using STAR/2.5.2a [55], sorted and indexed with samtools/1.9 [49], and then analyzed with MAJIQ/2.0 [56]. Splicing events were quantified for downstream analysis using the voila classify function, which groups splicing events into distinct modules. Events were considered significant if the probability of having a ΔPSI > 10 was greater than 90%.

### RT-PCR analysis of splicing

Validation of splicing events by low-cycle radiolabeled PCR was performed as previously described [57] with the following modifications. Annealing temperatures during PCR amplification, and the number of cycles required to maintain the signal in the linear range, were determined empirically for each splicing event. Gene specific primers (S1 Data) and were designed with the aid of MAJIQ’s voila visualization tool. We note that load controls were not used, and therefore overall abundance of the transcripts from each sample cannot be compared. However, the relative ratio between the transcript isoforms (bands) in each lane is internally controlled, which was used for quantifying alternative splicing.

### Immunoblot analyses

Immunoblot analysis was performed as described in [13]. Mouse anti-RNase L antibody 2E9 (Novus Biologicals catalog no. NB100-351) was used at 1:1500. Histone H3 antibody (Thermo Fisher Scientific; NB500-171) was used at 1:1000. Mouse anti-alpha tubulin (Abcam: ab18251) was used at 1:1000. Rabbit anti-GAPDH (Cell Signaling Technology: 2118L) was used at 1:2000. Anti-rabbit immunoglobulin G (IgG), horseradish peroxidase (HRP)–linked antibody (Cell Signaling Technology: 7074S) was used at 1:3000. Anti-mouse IgG, HRP-linked antibody (Cell Signaling Technology: 7076S) was used at 1:10,000 Crude nuclear and cytoplasmic fractionation was performed as described in [58].

### Immunofluorescence and smFISH

smFISH was performed as described in [13] and following the manufacturer’s protocol (https://biosearchassets.blob.core.windows.net/assets/bti_custom_stellaris_immunofluorescence_seq_protocol.pdf). GAPDH smFISH probes labeled with Quasar 570 dye (SMF-2026-1) were purchased from Stellaris. Custom IFNB1, IFNL1, SARS-CoV-2, and DENV2 smFISH probes [13,19] were designed using Stellaris smFISH probe designer (Biosearch Technologies) available online at http://biosearchtech.com/stellaris-designer. Reverse complement DNA oligos were purchased from IDT (S1 Data). The probes were labeled with ATTO-633 using ddUTP-Atto633 (Axxora: JBS-NU-1619-633), ATTO-550 using 5-Propargylamino-ddUTP (Axxora; JBS-NU-1619-550), or ATTO-488 using 5-Propargylamino-ddUTP (Axxora; JBS-NU-1619-488) with terminal deoxynucleotidyl transferase (Thermo Fisher Scientific: EP0161) as described in [59].

Antibodies used for immunofluorescence assays and their concentration are as follows. Rabbit polyclonal anti-PABPC1 antibody (Abcam: ab21060) was used at 1:1000. Rabbit polyclonal anti-HuR antibody (Proteintech: 11910-AP) was used at 1:500. Rabbit polyclonal anti-PABPC4 antibody (Thermo Scientific: 14960-1-AP) was used at 1:300. Rabbit anti-FAM120A (Sigma–Aldrich: HPA019734) was used at 1:500. Rabbit polyclonal anti-TIA1 (Abcam: ab40693) was used at 1:500. Rabbit polyclonal anti-Caprin1 (Fisher Scientific: 50–554–357) was used at 1:500. Rabbit polyclonal anti-PUM1 (Thermo Fisher Scientific: PA5-30327) was used at 1:500. Mouse anti-ataxin2 antibody (BD biosciences: 611378) was use at 1:500. Rabbit polyclonal anti-FMRP (Abcam: ab17722) was used at 1:500. Mouse monoclonal anti-G3BP antibody (Abcam: ab56574) was used at 1:1000. Goat anti-mouse IgG FITC (Abcam: ab97022) was used at 1:1000. Goat anti-rabbit IgG Alexa Fluor 647 (Abcam: ab150079) was used at 1:1000.

### Microscopy and image analysis

Coverslips were mounted on slides with VECTASHIELD Antifade Mounting Medium containing 4′,6-diamidino-2-phenylindole (DAPI) (Vector Laboratories; H-1200). Images were obtained using a wide-field DeltaVision Elite microscope with a 100× objective using a PCO Edge sCMOS camera. Between 10 and 15 Z planes at 0.2 μm per section were taken for each image for smFISH analyses. Five Z planes at 0.3 μm per section were taken for each image for immunofluorescence analyses. Deconvoluted images were processed using ImageJ with FIJI plugin. Z planes were stacked, and minimum and maximum display values were set in ImageJ for each channel to properly view fluorescence. Fluorescence intensity was measured in ImageJ. Single cells were outlined by determining the cell boundaries via background fluorescence and mean intensity was measured in the relevant channels. Imaris Image Analysis Software (Bitplane) (University of Colorado Boulder, BioFrontiers Advanced Light Microscopy Core) was used to quantify smFISH foci. Single cells were isolated for analysis by defining their borders via background fluorescence. Total foci above background threshold intensity were counted. Afterward, the nucleus marked with DAPI was masked, and foci were counted in the cell at the same intensity threshold cutoff, yielding the cytoplasmic foci count. For quantitative analyses, cells were analyzed in at least three fields of view from a single experiment in which all experimental and control samples were prepared simultaneously. Graphs generally display a single experimental replicate. However, several graphs show multiple replicates, which we note in the figure legends.

### Figure generation

The model figure was created with BioRender.com.

### Statistical analyses

P-values were derived either by one-way ANOVA with Tukey HSD (https://www.socscistatistics.com/tests/anova/default2.aspx) or student’s t-test (Excel). The specific test used for each figure is specified in each figure legend. * p<0.05, **p>0.01, ***p>0.001 unless otherwise noted in the figure legend.

## Supporting information

S1 Fig (A-C) IF for indicated proteins in WT and RL-KO A549 cells four following transfection with or without poly(I:C). (D) IF for indicated proteins in WT and RL-KO A549 cells four hours post-poly(I:C). (E) Quantification of the nuclear: cytoplasmic ratio of proteins shown in (D).
(TIFF)

S2 Fig (A) Number of splicing events with probability>0.9 for a |ΔPSI|>10%. (B) Dot plot of showing the ΔPSI (in fraction form) of splicing events in untreated vs poly(I:C)-treated WT and RL-KO cells. (C) Histograms showing the number of splicing events in each comparison binned by the probability that the |ΔPSI| > 10%
(TIFF)

S3 Fig (A) Distribution of intron/exon ratios in WT and RL-KO cells following mock or poly(I:C) lipofection of RNAs that are unchanged (padj > 0.5) in WT cells following poly(I:C) lipofection. B) Read coverage mapping to an unchanged RNA–TTC32. (C) RNAs that are downregulated (padj < 0.5, Log2Fold Change < -1). D) Read coverage mapping to a down-regulated RNA–TMEM60. (E) RNAs that are upregulated (padj < 0.5, Log2Fold Change > 1). F) Read coverage mapping to an up-regulated RNA–NOCT. Blue and red traces correspond to mapped reads on positive and negative strand respectively.
(TIFF)

S4 Fig (A) Distribution of DoG/exon ratios in WT and RL-KO cells following mock or poly(I:C) lipofection of RNAs that are unchanged (padj > 0.5) in WT cells following poly(I:C) lipofection. B) Read coverage mapping to an unchanged RNA–TTC32. (C) RNAs that are downregulated (padj < 0.5, Log2Fold Change < -1). D) Read coverage mapping to a down-regulated RNA–TMEM60. IGV trace scale was decreased 2.5x after autoscaling. (E) RNAs that are upregulated (padj < 0.5, Log2Fold Change > 1). F) Read coverage mapping to an up-regulated RNA–NOCT. Blue and red traces correspond to mapped reads on positive and negative strand respectively.
(TIFF)

S5 Fig (A) smFISH for GAPDH or ACTB mRNA in WT and RL-KO cells following poly(I:C) transfection. (B) IGV track of GAPDH and ACTB genes in WT and RL-KO cells post-poly(I:C).
(TIFF)

S6 Fig (A) IGV tracks for NORAD in WT and RL-KO cells. Below, smFISH probes targeting the NORAD RNA or DoG are shown. (B) smFISH for NORAD CDS and DoG RNA in WT or RL-KO cells twelve hours following mock transfection or transfection with poly(I:C).
(TIFF)

S7 Fig (A) smFISH for IFNB1-CDS and IFNB1-DoG sixteen hours post-lipofection of poly(I:C), as shown in Fig 4C. (B) quantification of IFNB1 CDS in WT and RL-KO cells as represented in (A). (C-E) similar to (A) and (B) except analyzing IFNL1 CDS, IFNL1 Dog-1, and IFNL1 intron.
(TIFF)

S8 Fig (A) Co-smFISH for IFNB1 CDS and DOG-2 region in A549 WT and RL-KO cells sixteen hours post-lipofection with poly(I:C). (B) similar to (A) but for IFNL1 CDS and DoG-2 region.
(TIFF)

S9 Fig (A) smFISH for IFNB CDS and DoG-1 in WT and RL-KO cells, as well as RL-KO cells stably expressing either RNase L or RNase L-R667A (catalytic mutant) twelve hours after transfection with poly(I:C). (B) Quantification of IFNB-DoG smFISH foci as represented in (A).
(TIFF)

S10 Fig (A) smFISH for IFNB1-CDS and IFNB1-DoG eight hours post-lipofection of poly(I:C). Red arrows indicate cells that contain high levels of both nuclear-retained IFNB1-CDS and IFNB1-DoG foci that mostly co-localize. White arrows indicate cells that do not contain high levels of IFNB1-DoG foci, which remain largely localized the IFNB1 genomic loci, and IFNB1-CDS is primarily localized to the cytoplasm. Yellow arrows demarcate cells that contain high levels of nuclear-localized IFNB1-CDS but not IFNB1-DoG foci. (B) Box plots displaying of the number of IFNB1-DoG foci localized to the nucleus or cytoplasm in WT or RL-KO cells as represented in (A). Scatter plot of the ratio (nucleus/cytoplasm) of IFNB1-CDS foci (x-axis) and nuclear IFNB1-DoG foci in WT and RL-KO cells as represented in (A). (D) Scatter plots of the quantity of nuclear IFNB1-DoG foci (y-axis) and the quantity of nuclear IFNB1-CDS foci (x-axis) in WT and RL-KO cells as represented in (A). (E) smFISH for IFNL1-CDS and IFNL1-DoG in WT or RL-KO eight hours post-poly(I:C) lipofection.
(TIFF)

S11 Fig (A) smFISH for GAPDH and DENV2 mRNAs in WT A549 cells 48 hours post-infection with DENV2 (MOI = 0.1). (B) similar to (A) but staining for PABP via IF.
(TIFF)

S12 Fig (A) Quantification of the percent of *IFNB1* mRNA smFISH foci in the nucleus of A549 cells expressing either mRuby2-PABPC1 or mRuby2-PABPC1-NRS eight hours post-poly(I:C). Between 17–31 cells were analyzed from two independent experiments. (B) Nucleus to cytoplasm ratio of PABP fluorescence intensity in A549 cells forty-eight hours post-transfection of control siRNA (siNC) or siRNAs targeting XRN1 (siXRN1). Between 14–19 cells were analyzed from two independent experiments. (C) Quantification of total and nuclear IFNB1-CDS or IFNB1-DoG smFISH foci in A549 cells transfected with siNC or siXRN1 siRNAs eight hours post-transfection of poly(I:C). Between 16–28 cells were analyzed from two independent experiments. Statistical significance was determined using student’s ttest. *< 0.05, *
(TIFF)

S1 Data Lists of gene blocks, RT-qPCR primers, smFISH oligos, and spread sheets for data analyses. (XLSX)
