# Supplementary figures and images for: RNase L activation in the cytoplasm induces aberrant processing of mRNAs in the nucleus
Source: PLoS Pathog. 2022 Nov 1;18(11):e1010930. doi: 10.1371/journal.ppat.1010930 (PMC9651596; doi:10.1371/journal.ppat.1010930)

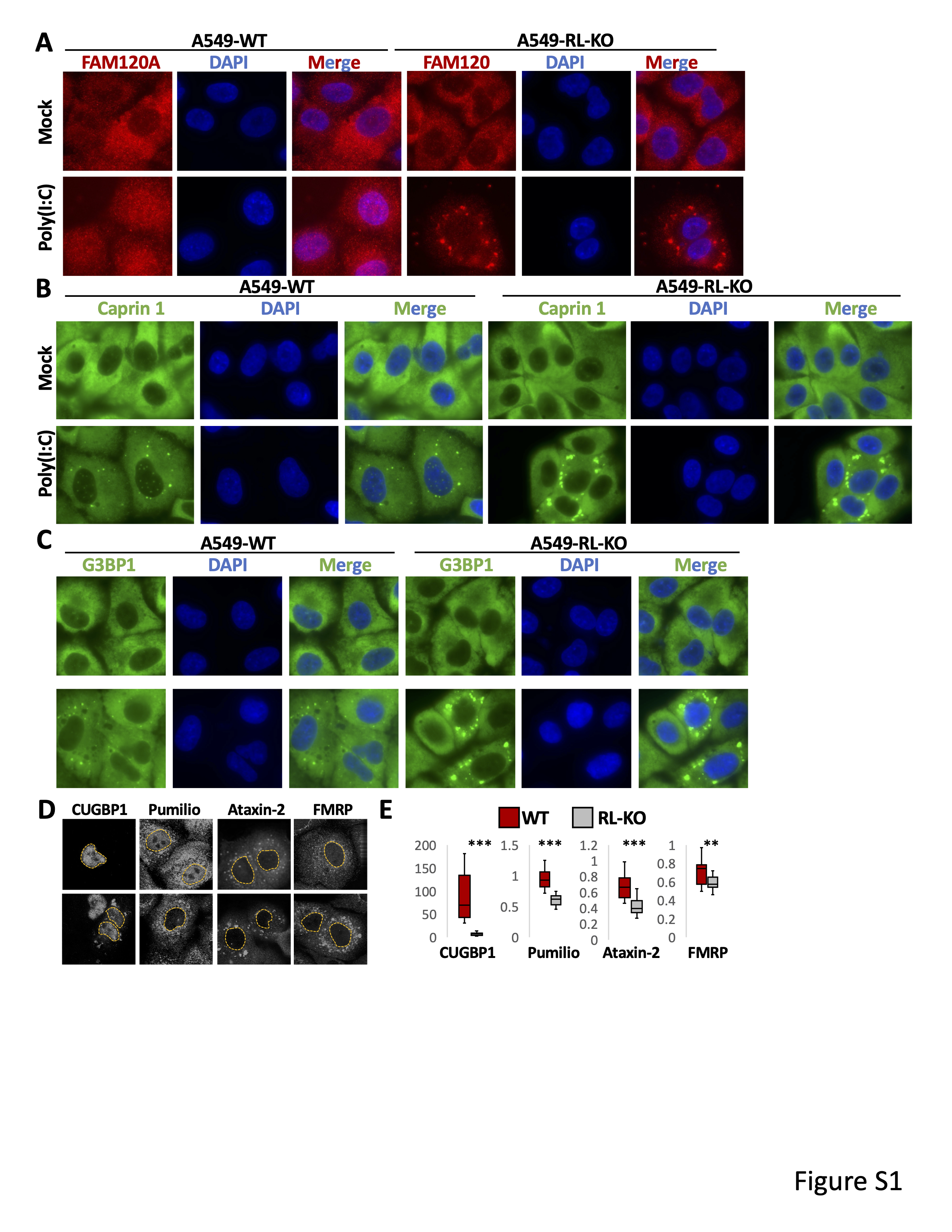

Supplement: S1 Fig — (A-C) IF for indicated proteins in WT and RL-KO A549 cells four following transfection with or without poly(I:C). (D) IF for indicated proteins in WT and RL-KO A549 cells four hours post-poly(I:C). (E) Quantification of the nuclear: cytoplasmic ratio of proteins shown in (D). (TIFF) [file ppat.1010930.s001.tiff]

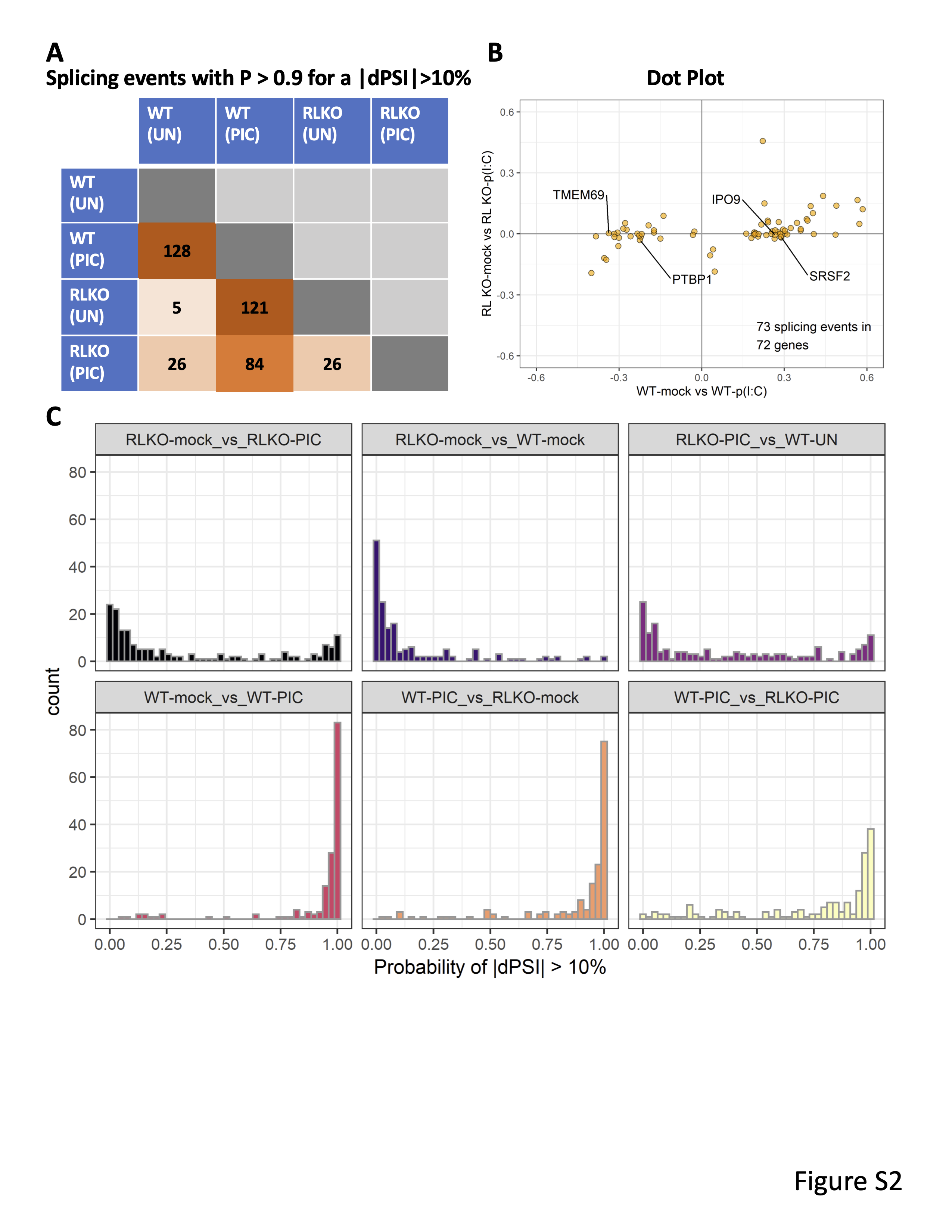

Supplement: S2 Fig — (A) Number of splicing events with probability>0.9 for a |ΔPSI|>10%. (B) Dot plot of showing the ΔPSI (in fraction form) of splicing events in untreated vs poly(I:C)-treated WT and RL-KO cells. (C) Histograms showing the number of splicing events in each comparison binned by the probability that the |ΔPSI| > 10% (TIFF) [file ppat.1010930.s002.tiff]

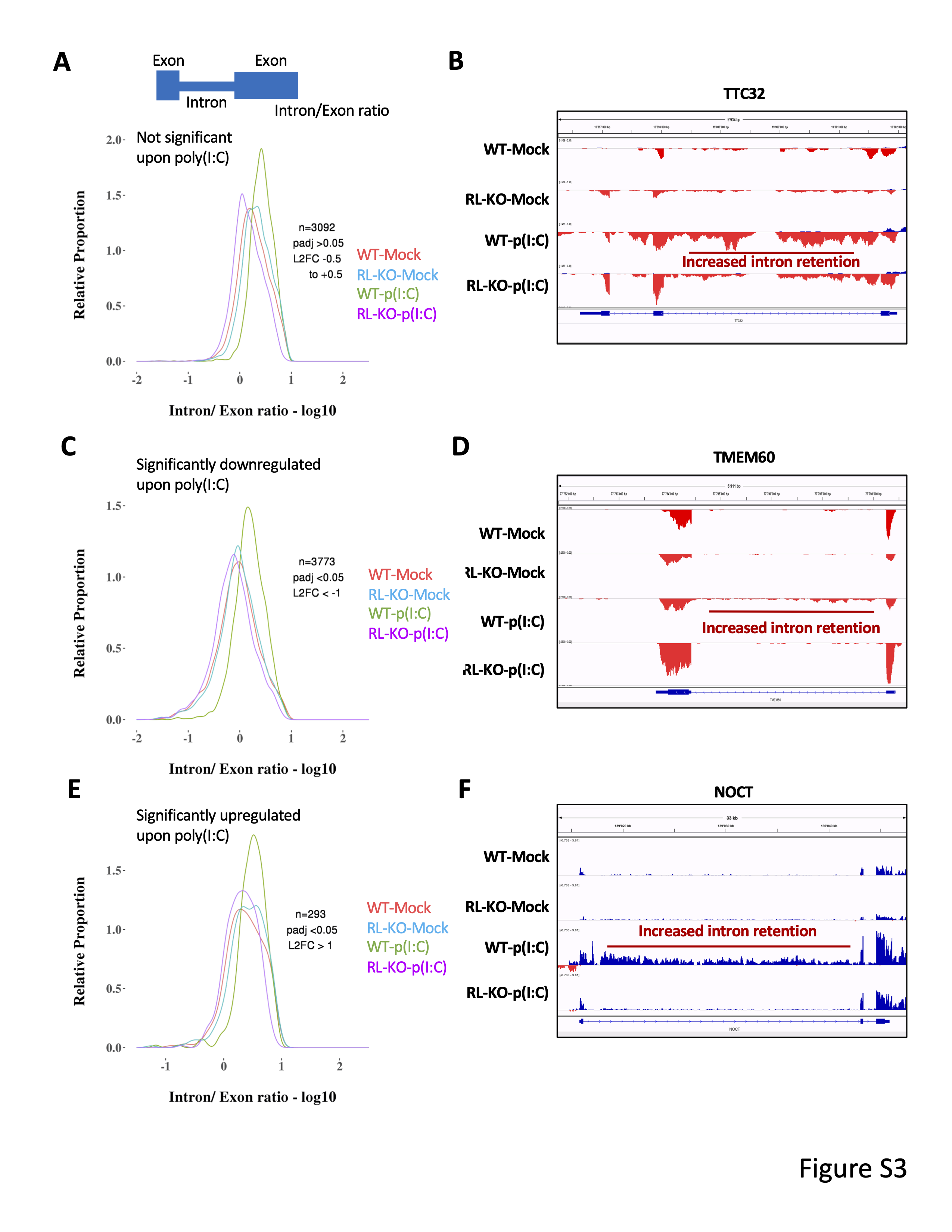

Supplement: S3 Fig — (A) Distribution of intron/exon ratios in WT and RL-KO cells following mock or poly(I:C) lipofection of RNAs that are unchanged (padj > 0.5) in WT cells following poly(I:C) lipofection. B) Read coverage mapping to an unchanged RNA–TTC32. (C) RNAs that are downregulated (padj < 0.5, Log2Fold Change < -1). D) Read coverage mapping to a down-regulated RNA–TMEM60. (E) RNAs that are upregulated (padj < 0.5, Log2Fold Change > 1). F) Read coverage mapping to an up-regulated RNA–NOCT. Blue and red traces correspond to mapped reads on positive and negative strand respectively. (TIFF) [file ppat.1010930.s003.tiff]

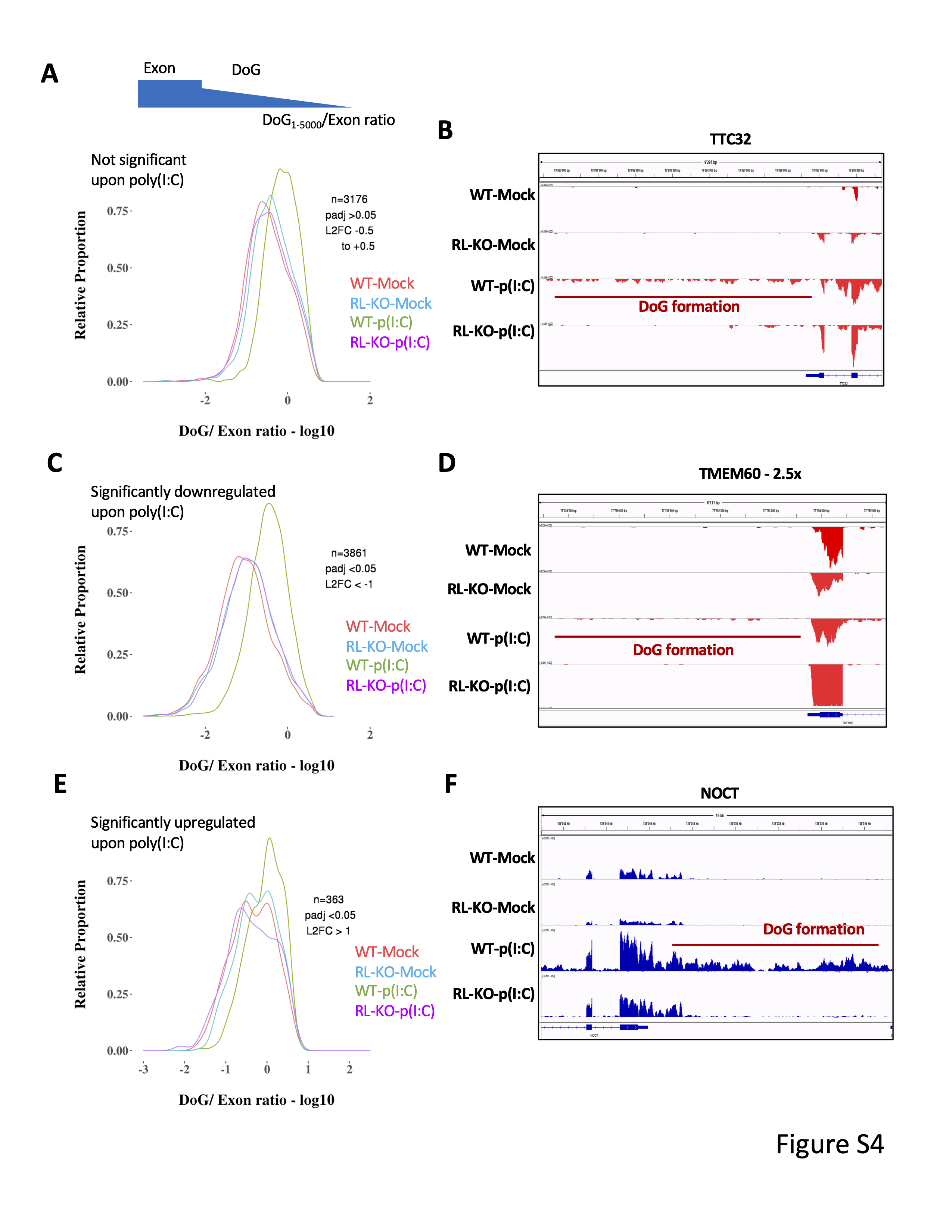

Supplement: S4 Fig — (A) Distribution of DoG/exon ratios in WT and RL-KO cells following mock or poly(I:C) lipofection of RNAs that are unchanged (padj > 0.5) in WT cells following poly(I:C) lipofection. B) Read coverage mapping to an unchanged RNA–TTC32. (C) RNAs that are downregulated (padj < 0.5, Log2Fold Change < -1). D) Read coverage mapping to a down-regulated RNA–TMEM60. IGV trace scale was decreased 2.5x after autoscaling. (E) RNAs that are upregulated (padj < 0.5, Log2Fold Change > 1). F) Read coverage mapping to an up-regulated RNA–NOCT. Blue and red traces correspond to mapped reads on positive and negative strand respectively. (TIFF) [file ppat.1010930.s004.tiff]

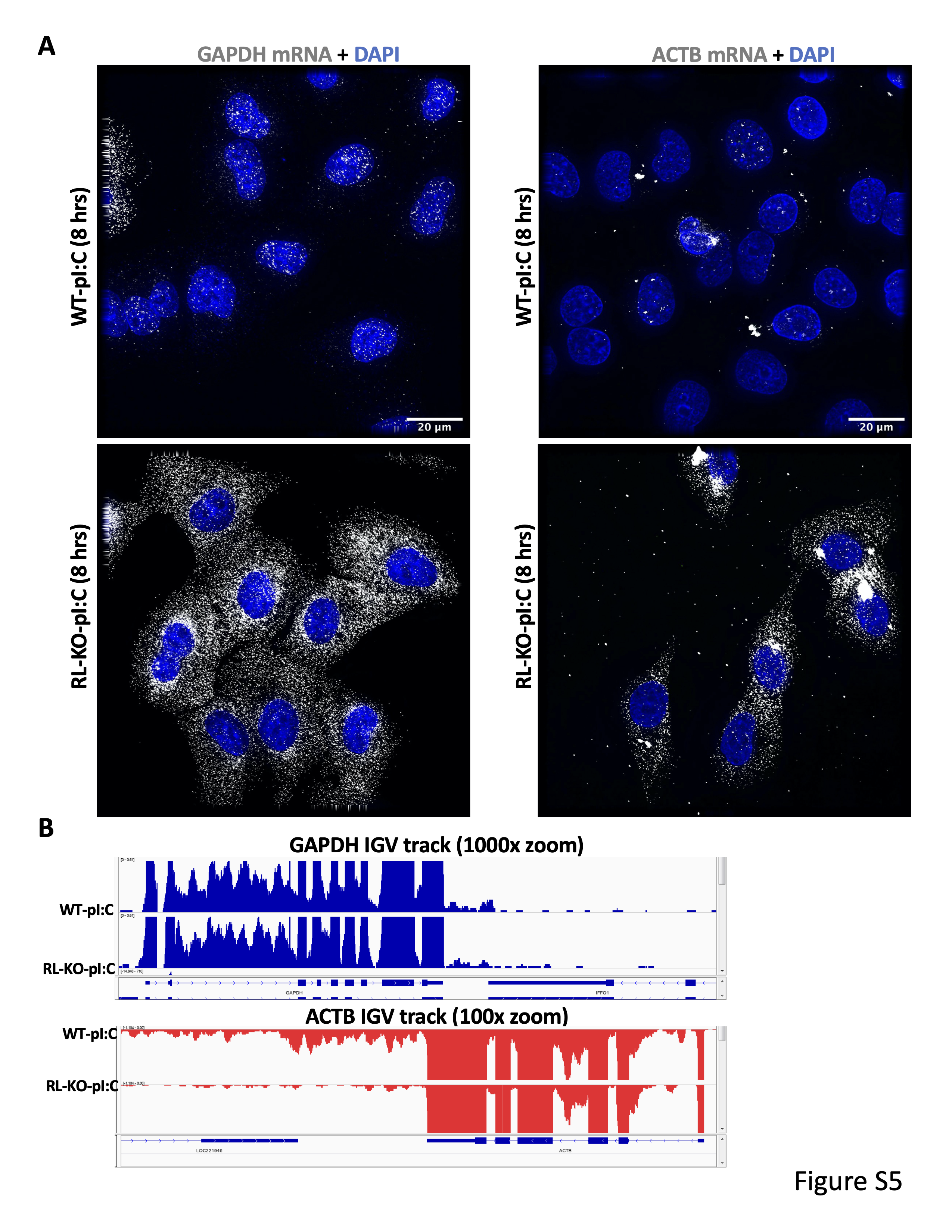

Supplement: S5 Fig — (A) smFISH for GAPDH or ACTB mRNA in WT and RL-KO cells following poly(I:C) transfection. (B) IGV track of GAPDH and ACTB genes in WT and RL-KO cells post-poly(I:C). (TIFF) [file ppat.1010930.s005.tiff]

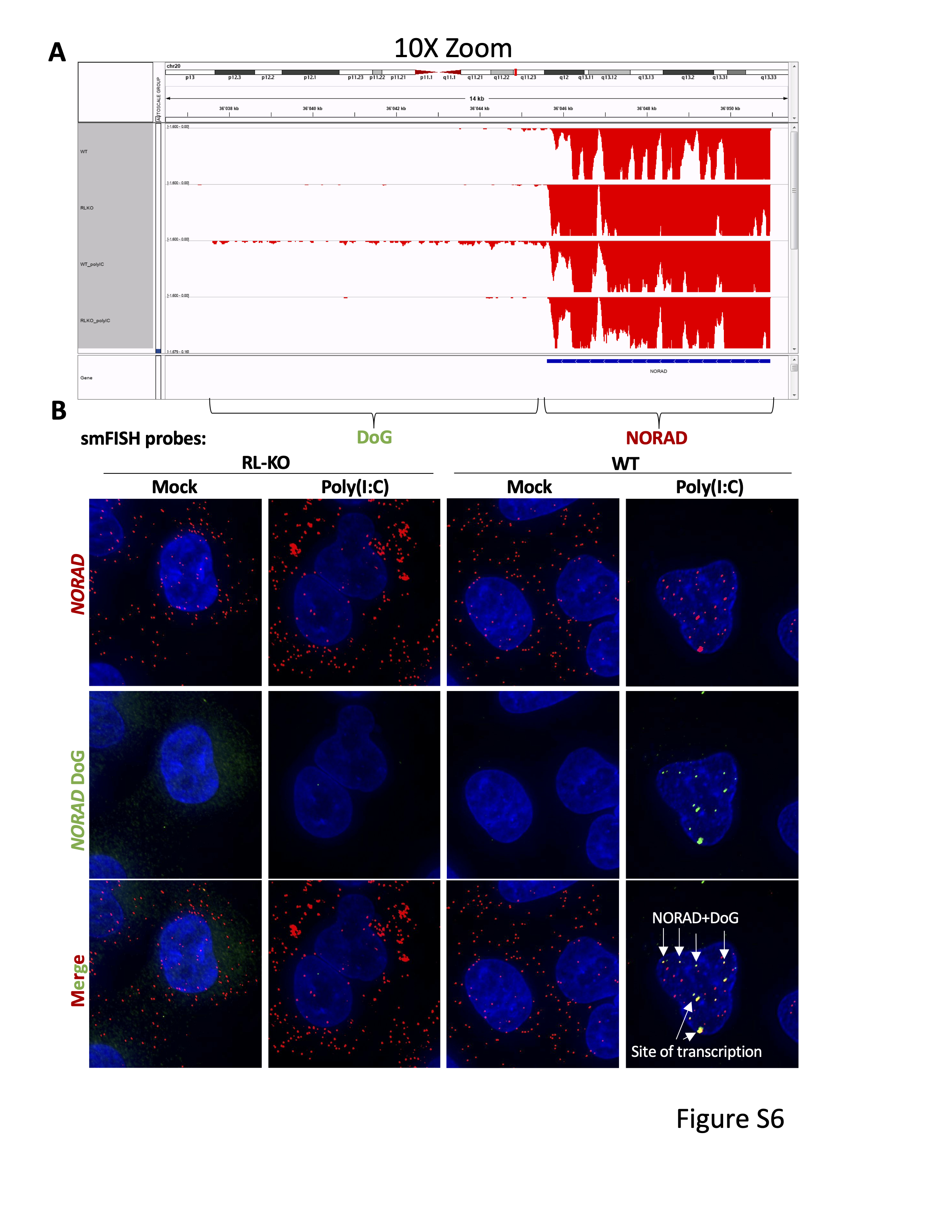

Supplement: S6 Fig — (A) IGV tracks for NORAD in WT and RL-KO cells. Below, smFISH probes targeting the NORAD RNA or DoG are shown. (B) smFISH for NORAD CDS and DoG RNA in WT or RL-KO cells twelve hours following mock transfection or transfection with poly(I:C). (TIFF) [file ppat.1010930.s006.tiff]

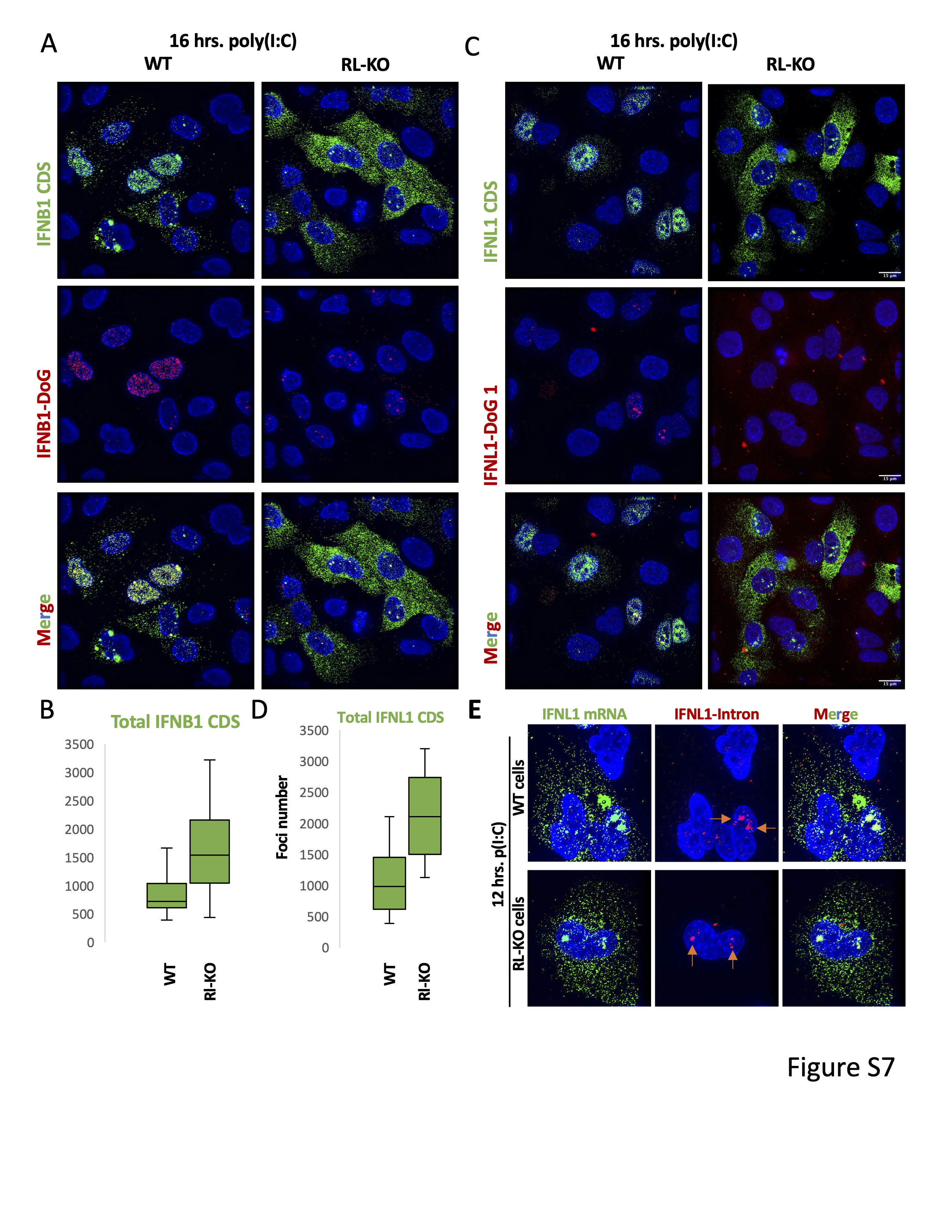

Supplement: S7 Fig — (A) smFISH for IFNB1-CDS and IFNB1-DoG sixteen hours post-lipofection of poly(I:C), as shown in Fig 4C. (B) quantification of IFNB1 CDS in WT and RL-KO cells as represented in (A). (C-E) similar to (A) and (B) except analyzing IFNL1 CDS, IFNL1 Dog-1, and IFNL1 intron. (TIFF) [file ppat.1010930.s007.tiff]

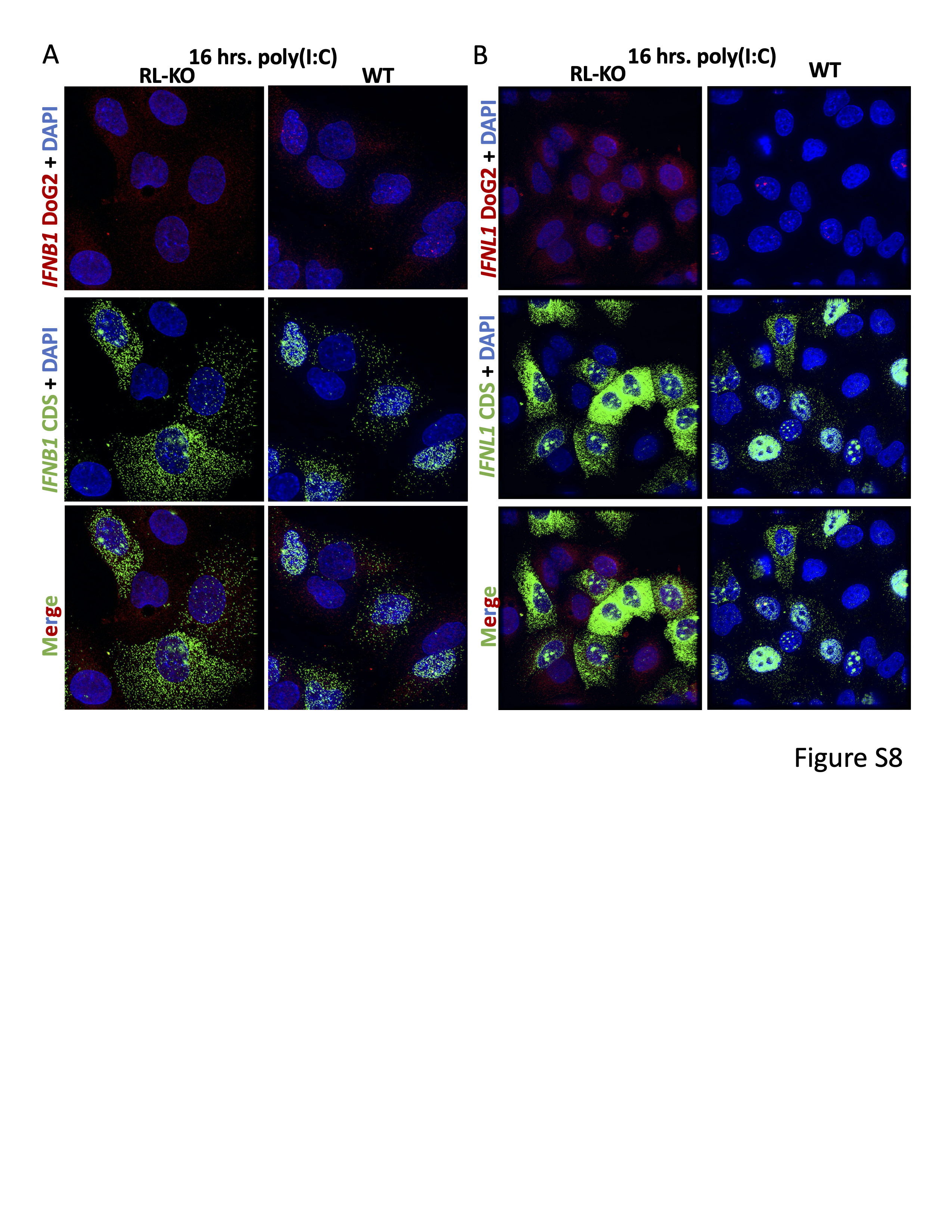

Supplement: S8 Fig — (A) Co-smFISH for IFNB1 CDS and DOG-2 region in A549 WT and RL-KO cells sixteen hours post-lipofection with poly(I:C). (B) similar to (A) but for IFNL1 CDS and DoG-2 region. (TIFF) [file ppat.1010930.s008.tiff]

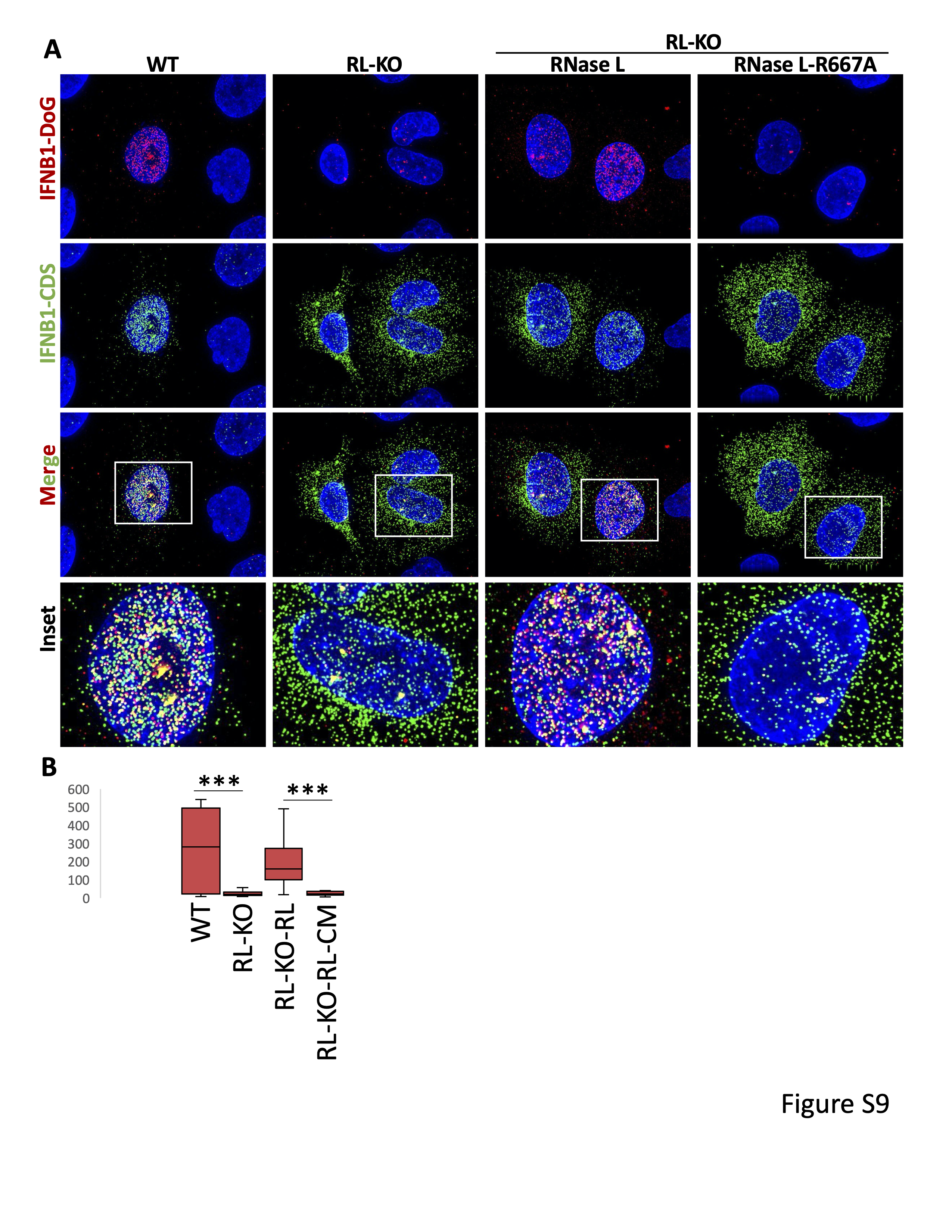

Supplement: S9 Fig — (A) smFISH for IFNB CDS and DoG-1 in WT and RL-KO cells, as well as RL-KO cells stably expressing either RNase L or RNase L-R667A (catalytic mutant) twelve hours after transfection with poly(I:C). (B) Quantification of IFNB-DoG smFISH foci as represented in (A). (TIFF) [file ppat.1010930.s009.tiff]

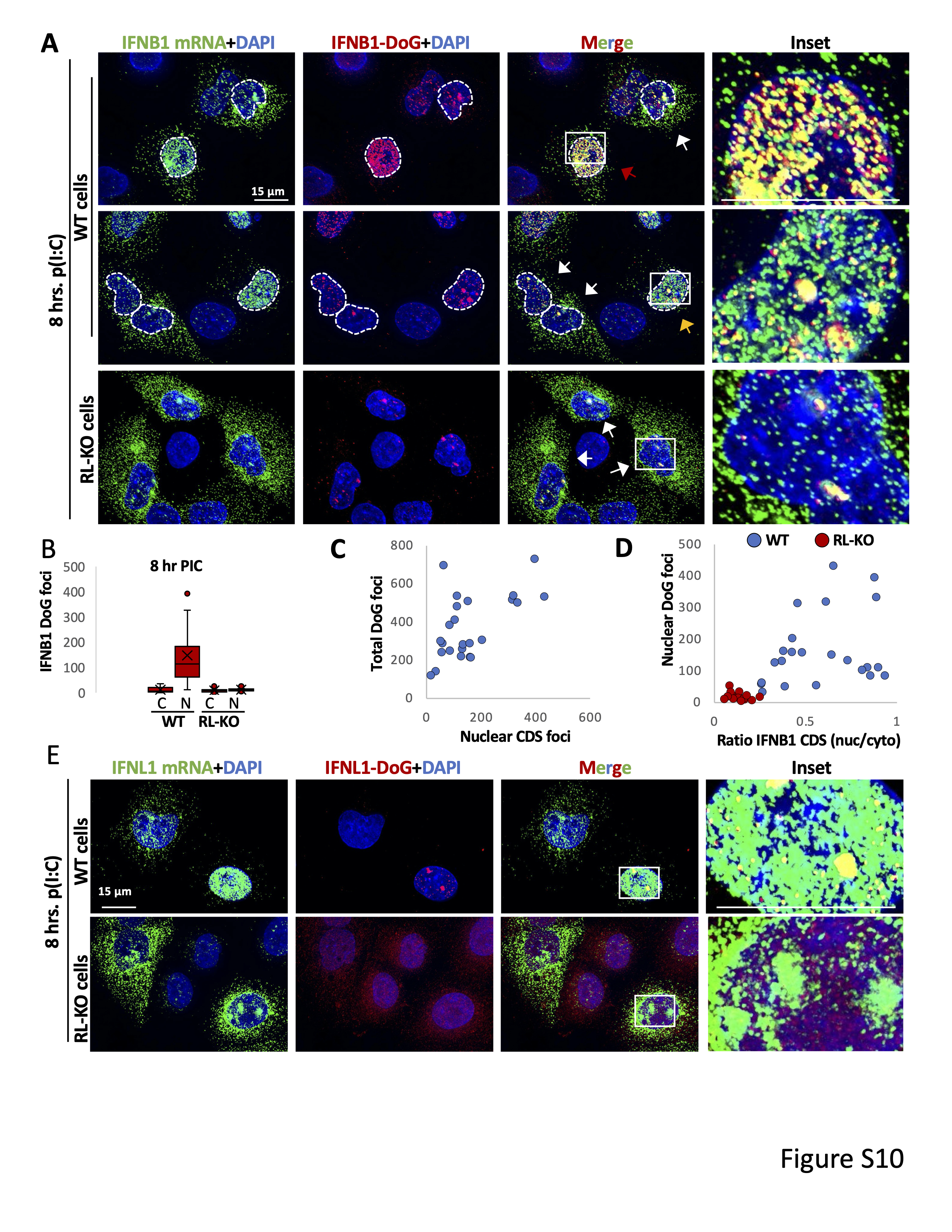

Supplement: S10 Fig — (A) smFISH for IFNB1-CDS and IFNB1-DoG eight hours post-lipofection of poly(I:C). Red arrows indicate cells that contain high levels of both nuclear-retained IFNB1-CDS and IFNB1-DoG foci that mostly co-localize. White arrows indicate cells that do not contain high levels of IFNB1-DoG foci, which remain largely localized the IFNB1 genomic loci, and IFNB1-CDS is primarily localized to the cytoplasm. Yellow arrows demarcate cells that contain high levels of nuclear-localized IFNB1-CDS but not IFNB1-DoG foci. (B) Box plots displaying of the number of IFNB1-DoG foci localized to the nucleus or cytoplasm in WT or RL-KO cells as represented in (A). Scatter plot of the ratio (nucleus/cytoplasm) of IFNB1-CDS foci (x-axis) and nuclear IFNB1-DoG foci in WT and RL-KO cells as represented in (A). (D) Scatter plots of the quantity of nuclear IFNB1-DoG foci (y-axis) and the quantity of nuclear IFNB1-CDS foci (x-axis) in WT and RL-KO cells as represented in (A). (E) smFISH for IFNL1-CDS and IFNL1-DoG in WT or RL-KO eight hours post-poly(I:C) lipofection. (TIFF) [file ppat.1010930.s010.tiff]

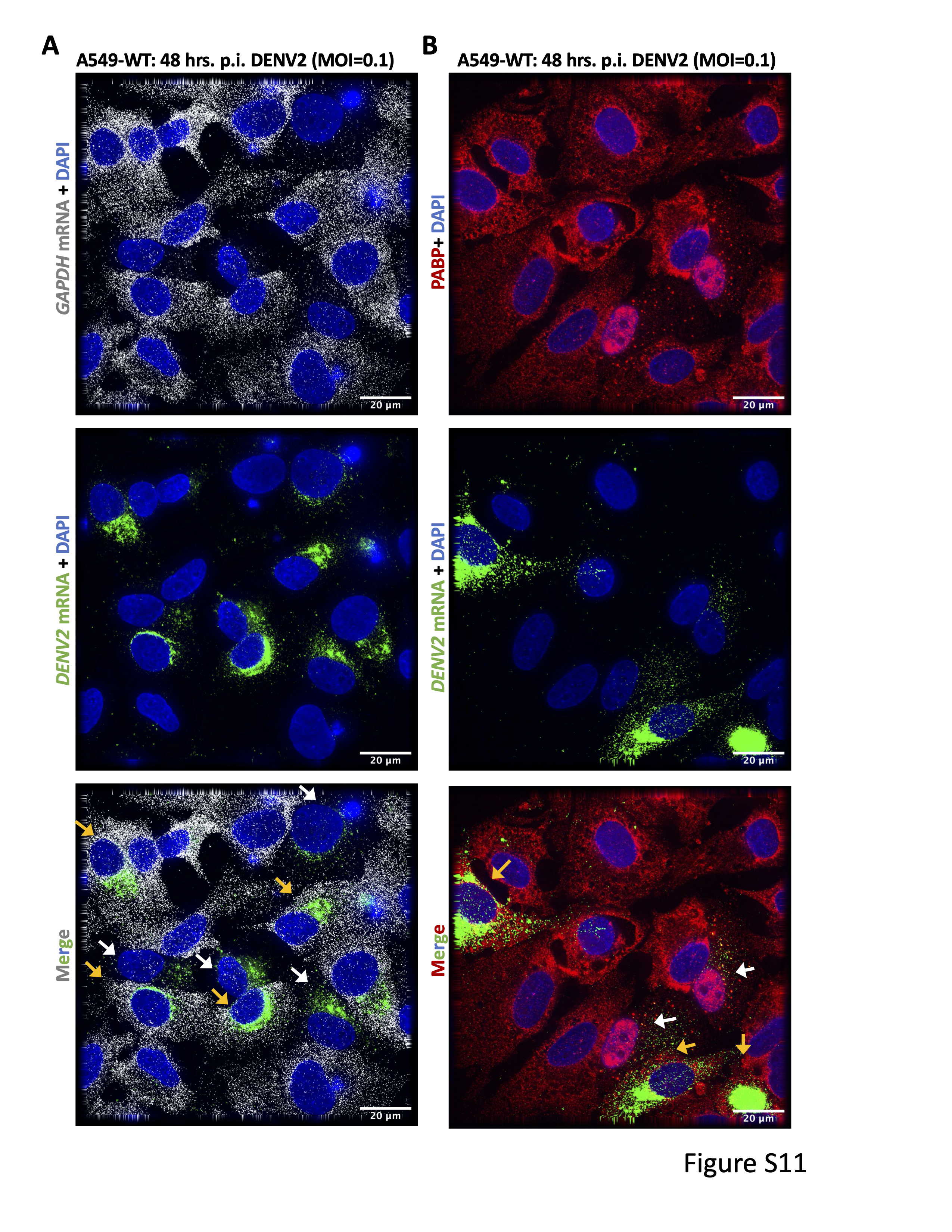

Supplement: S11 Fig — (A) smFISH for GAPDH and DENV2 mRNAs in WT A549 cells 48 hours post-infection with DENV2 (MOI = 0.1). (B) similar to (A) but staining for PABP via IF. (TIFF) [file ppat.1010930.s011.tiff]

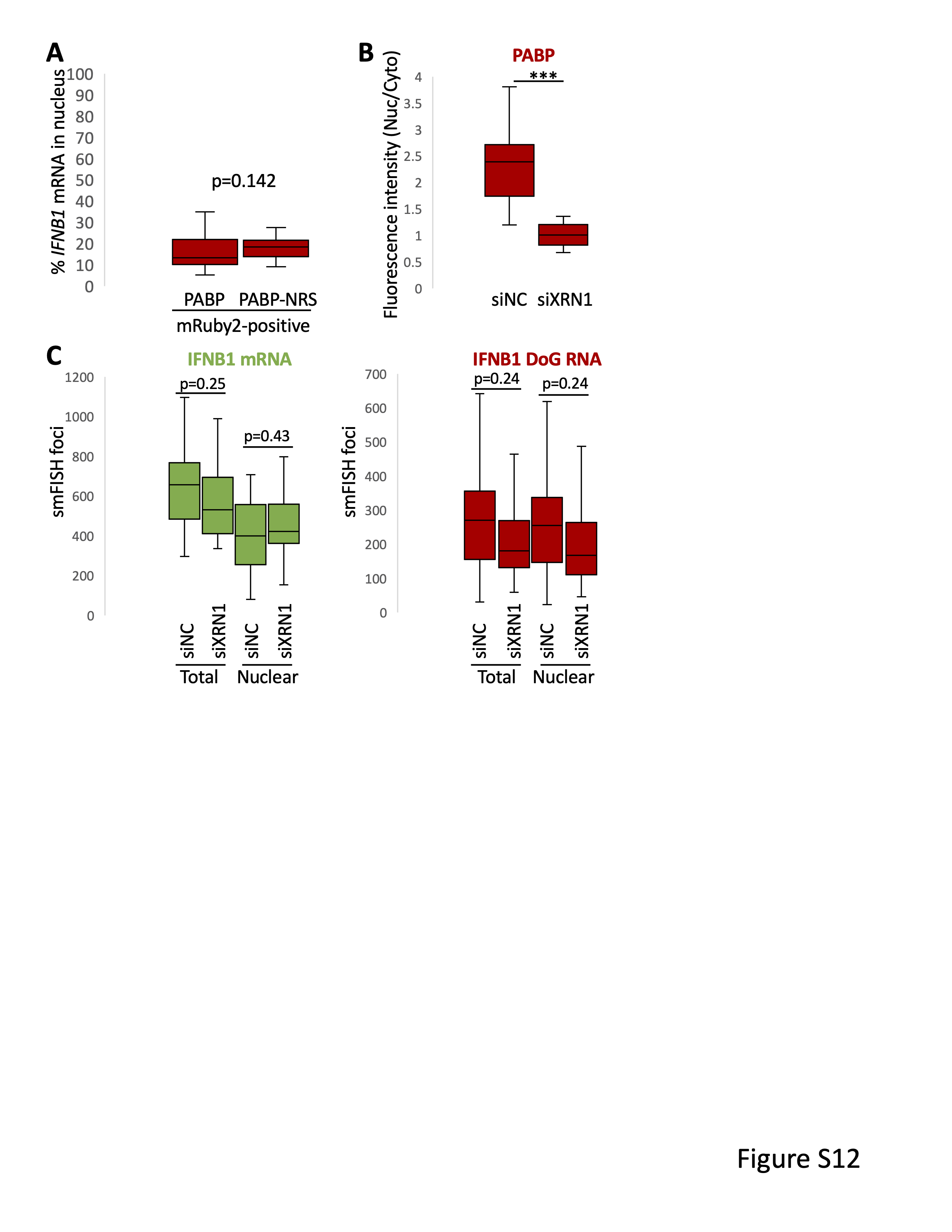

Supplement: S12 Fig — (A) Quantification of the percent of IFNB1 mRNA smFISH foci in the nucleus of A549 cells expressing either mRuby2-PABPC1 or mRuby2-PABPC1-NRS eight hours post-poly(I:C). Between 17–31 cells were analyzed from two independent experiments. (B) Nucleus to cytoplasm ratio of PABP fluorescence intensity in A549 cells forty-eight hours post-transfection of control siRNA (siNC) or siRNAs targeting XRN1 (siXRN1). Between 14–19 cells were analyzed from two independent experiments. (C) Quantification of total and nuclear IFNB1-CDS or IFNB1-DoG smFISH foci in A549 cells transfected with siNC or siXRN1 siRNAs eight hours post-transfection of poly(I:C). Between 16–28 cells were analyzed from two independent experiments. Statistical significance was determined using student’s ttest. *< 0.05, * (TIFF) [file ppat.1010930.s012.tiff]
